# The prevalence of premenstrual syndrome in China: a systematic review and meta-analyses

**DOI:** 10.3389/fpsyt.2025.1640781

**Published:** 2025-09-11

**Authors:** Yanmei Yang, Yu Jia, Songying Fu, Lu Zhang, Feng Xiang, Wanqin Hu, Xuehua Cao

**Affiliations:** ^1^ School of Nursing, Chengdu University of Traditional Chinese Medicine, Sichuan, China; ^2^ Department of Gynecology Nursing, Sichuan Provincial People’s Hospital, University of Electronic Science and Technology of China, Sichuan, China

**Keywords:** premenstrual syndrome, China, prevalence, women, systematic review

## Abstract

**Background:**

Reliable estimates of the prevalence of premenstrual syndrome (PMS) serve as the basis for adequate prevention and treatment. However, the prevalence of PMS has rarely been synthesized at the Chinese level. We conducted this systematic review and meta-analyses to provide accurate and comprehensive evidence on the prevalence of PMS in China

**Methods:**

PubMed, Web of Science, Embase, Cochrane Library, China National Knowledge Information, Chinese Scientific Journal (VIP), Wan fang and China Biology Medicine databases were systematically searched from inception until March 8, 2025. The prevalence of PMS in China was analyzed using a random effects model.

**Results:**

A total of 77 studies (108,178 participants) were included in the meta-analyses. The pooled prevalence of PMS in China was 48% (95% CI, 44-53%). Subgroup analyses revealed significant differences in the prevalence of PMS according to the screening tool, region, setting, and mean age. Meta-regression analyses indicated that using a prospective tool was significantly associated with lower prevalence.

**Limitations:**

There was a high degree of heterogeneity among the included studies; The included studies were conducted in urban areas; There was significant publication bias in this meta - analyses; Current meta-analyses contain insufficient information on many risk factors for PMS in China.

**Conclusion:**

The findings demonstrate that most Chinese women have PMS. Given the prevalence of PMS in China, future studies should explore its risk and protective factors, provide regular screening, and implement effective preventive and treatment measures for this demographic.

## 1 Introduction

The American College of Obstetricians and Gynecologists (ACOG) defines premenstrual syndrome (PMS) as a clinical condition characterized by cyclical affective and somatic symptoms. These symptoms must be severe enough to cause dysfunction in social, academic, or work performance and unrelated to any organic disease (1). The diagnostic criteria established by ACOG require the presence of at least one affective symptom (including angry outbursts, anxiety, confusion, depression, irritability or social withdrawal) and one somatic symptom (including abdominal bloating, breast tenderness or swelling, headache, joint or muscle pain, swelling of extremities or weight gain), with mandatory temporal characteristics symptoms must appear during the late luteal phase, resolve within four days after the onset of menstruation, and occur in three consecutive menstrual cycles (1). These diagnostic criteria are consistent with the core criteria for PMS established by the International Society for Premenstrual Disorders (ISPMD) (2). Premenstrual dysphoric disorder (PMDD), a severe subtype of PMS, is diagnosed based on the criteria from the Diagnostic and Statistical Manual–fifth edition (DSM-5) of the American Psychiatric Association (APA) (3). These criteria require the presence of five symptoms in total, including at least one core affective symptom (mood swings, marked irritability, marked depressed mood or marked anxiety), with the affective symptoms severe enough to interfere with the ability to function. This is the key distinguishing feature between PMDD and PMS (4).

PMS is common among women of childbearing age (5). Up to 90% of women experienced at least one PMS symptom during their menstrual cycle, and approximately 20-40% of women meet the ACOG criteria for a PMS diagnosis (6). Symptoms often fluctuate in terms of both persistence and severity. studies found that only 36% of women diagnosed with PMS continued to meet diagnostic criteria one year later (7). Fewer women meet the more rigorous diagnostic criteria for PMDD, with prevalence ranged from 1.2-6.4% (8).

The impact of PMS on women’s health is multidimensional. Studies have shown that women with PMS not only face reduced health-related quality of life (9) but also lead to other adverse consequences, including more frequent hospital visits, decreased work productivity, and impaired interpersonal relationships (10–12). Notably, patients with PMS are at a significantly increased risk of comorbid psychiatric disorders, particularly perimenopausal depression, postpartum depression, and anxiety disorders (13).

Previous studies have demonstrated a dramatic 46.5% increase in the prevalence of PMS, from 652.5 million in 1990 to 956 million in 2019, affecting nearly half of the female population worldwide (14). Among the Chinese population, the Disability-Adjusted Life Years (DALYs) attributed to PMS amounts to 815,004.64 person-years, the highest among gynecological diseases (15). Reflecting that Chinese women are burdened with a heavy disease load related to PMS. This phenomenon may be linked to factors such as China’s unique cultural background and differences in health perceptions (16).

From the public health perspective, reliable estimates of the prevalence of premenstrual syndrome serve as the basis for adequate prevention and treatment, as well as evidence-based health resource allocation and policy making. Despite a considerable number of studies assessing the prevalence of PMS, there were significant differences in the prevalence reported between studies, ranging from 8.6% to 86.52% (17, 18). To our knowledge, the prevalence estimates of PMS have rarely been synthesized at the Chinese level. To fill this knowledge gap, we systematically reviewed studies that reported the prevalence of PMS in China. This study aimed to determine the prevalence of PMS in China through systematic review and meta-analyses.

## 2 Materials and methods

Our review was conducted in accordance with the Preferred Reporting Items for Systematic Reviews and Meta-Analyses (PRISMA) reporting guidelines (19). It has also been registered at PROSPERO (Registration No: CRD420251016643).

### 2.1 Inclusion and exclusion criteria

Based on the PICOS acronym, the following inclusion criteria were used: Participants (P): Chinese women (This includes women in menarche and premenopausal.); Interventions (I): not applicable; Comparison (C): not applicable; Outcome (O): prevalence of PMS or relevant data that would yield an estimate of PMS prevalence; Study design (S): cross-sectional study.

Exclusion criteria: a: syntheses, systematic review or duplicate publications; b: studies for which full text was unavailable, or data could not be converted; c: Studies that did not use ACOG or ISPMD diagnostic criteria. When multiple studies were reported in the same sample, we used the survey with the largest sample size.

### 2.2 Research search and selection

Systematic searches were conducted for studies published in PubMed, Web of Science, Embase, Cochrane Library, China National Knowledge Information, Chinese Scientific Journal (VIP), Wan Fang and China Biology Medicine databases for studies published in the timeframe from library construction to March 8 2025, and the final search strategy was developed by combining MeSH terms and related keywords (see **Supplementary Table 1**).

Two investigators independently screened the titles and abstracts according to the predefined inclusion and exclusion criteria to identify potentially eligible studies for full-text review. Subsequently, the two researchers performed cross-verification to determine the final eligible studies. In cases of disagreement, a third investigator (XHC) was consulted to facilitate resolution.

### 2.3 Data extraction and study quality assessment

Two researchers developed and used structured forms to extract data independently from the included studies, with any discrepancies resolved through discussion. Extracted data included first author, year of publication, study period, region, sample size, screening tool, mean age, prevalence of PMS, and data required for methodological quality assessment.

All studies included among this review were cross-sectional, so the methodological quality of the eligible studies was assessed using the evaluation tool developed by the Agency of Healthcare and Quality (AHRQ) (20). The AHRQ has 11 items, scoring 1 when an individual item is assessed as ‘yes’ and 0 when it is ‘no’ or ‘unclear’. Scores of 0-3, 4–7 and 8–11 were classified as low, moderate and high quality, respectively. Quality assessment is carried out by YMY, which YJ checks and any disagreements are resolved through discussion.

### 2.4 Statistical analyses

All statistical analyses were carried out using R Statistical Software version 4.3.3. Given the heterogeneity in screening tools and sample characteristics of the studies included, the pooled prevalence and 95% confidence intervals (CIs) for PMS were calculated using random effects models. Meanwhile, the *I* ^2^ statistic was used to assess study heterogeneity, which was considered significant heterogeneity when *I* ^2^≥50%. Publication bias was assessed using funnel plots and Egger’s test, while sensitivity analyses were used to test the stability of the main results. The statistical significance level was set at *P*< 0.05 (two-tailed).

/Subgroup and meta-regression analyses were used to explore potential sources of heterogeneity. Subgroup analyses based on the following categorical variables: region (North, South, Central, Southwest, Northwest, Northeast, and East China), screening tool (Premenstrual Syndrome Scale, PMSS; Premenstrual Symptoms Screening Tool, PSST; Menstrual Health Questionnaire, MHQ; Menstrual Symptom Questionnaire, MSQ; Daily Record of Severity of Problems, DRSP; Calendar of Premenstrual Experiences, COPE; Menstrual Distress Questionnaire, MDQ; Shortened Premenstrual Assessment Form, SPAF; Premenstrual Syndrome Diary, PMSD), study period, those that used validated tools and those that either did not report which questionnaire was used, or who used their author-developed questionnaire or non-validated tools, setting (educational site, community, workplace, or hospital), whether validated prospective tools was used. Meta-regression analyses were performed to examine the effect of the following variables on the prevalence of PMS when these variables were >10 studies: mean age, sample size, screening tool, study quality, setting, region, and whether a validated prospective tool was used.

### 2.5 Tools and validity

Among the evaluated scales, COPE, PSST, PMSS, SPAF, and MDQ have been validated in Chinese populations, whereas MSQ, MHQ, and PMSD lack local validation data.

The DRSP is a diagnostic instrument that meets the diagnostic criteria for PMS and can prospectively record PMS symptoms (21). Validation in the Chinese women showed a reliability and validity of 0.97 (22). The COPE, a validated and reliable PMS prospective assessment tool (23), with demonstrated applicability in Chinese populations (24). The PSST, based on DSM-IV criteria, identifies moderate to severe PMS and PMDD (25). Cross-cultural validation studies in Brazil and Iran have confirmed its universality (26, 27), with high reliability (0.92) in Chinese university students (17). The PMSS, is a widely utilized PMS screening scale that encompasses the fundamental affective and somatic symptoms delineated by the ACOG standard (28). Its reliability in the Chinese women has been validated at 0.80 (29). The SPAF (30), a simplified screening tool based on the Premenstrual Assessment Form (PAF) (31), with a reliability of 0.89 in the Chinese population (32). The MDQ, a screening tool for PMS symptoms (33), has a reliability of 0.937 in Chinese women among multicenter study (34).

## 3 Results

### 3.1 Search results

**Figure 1:**
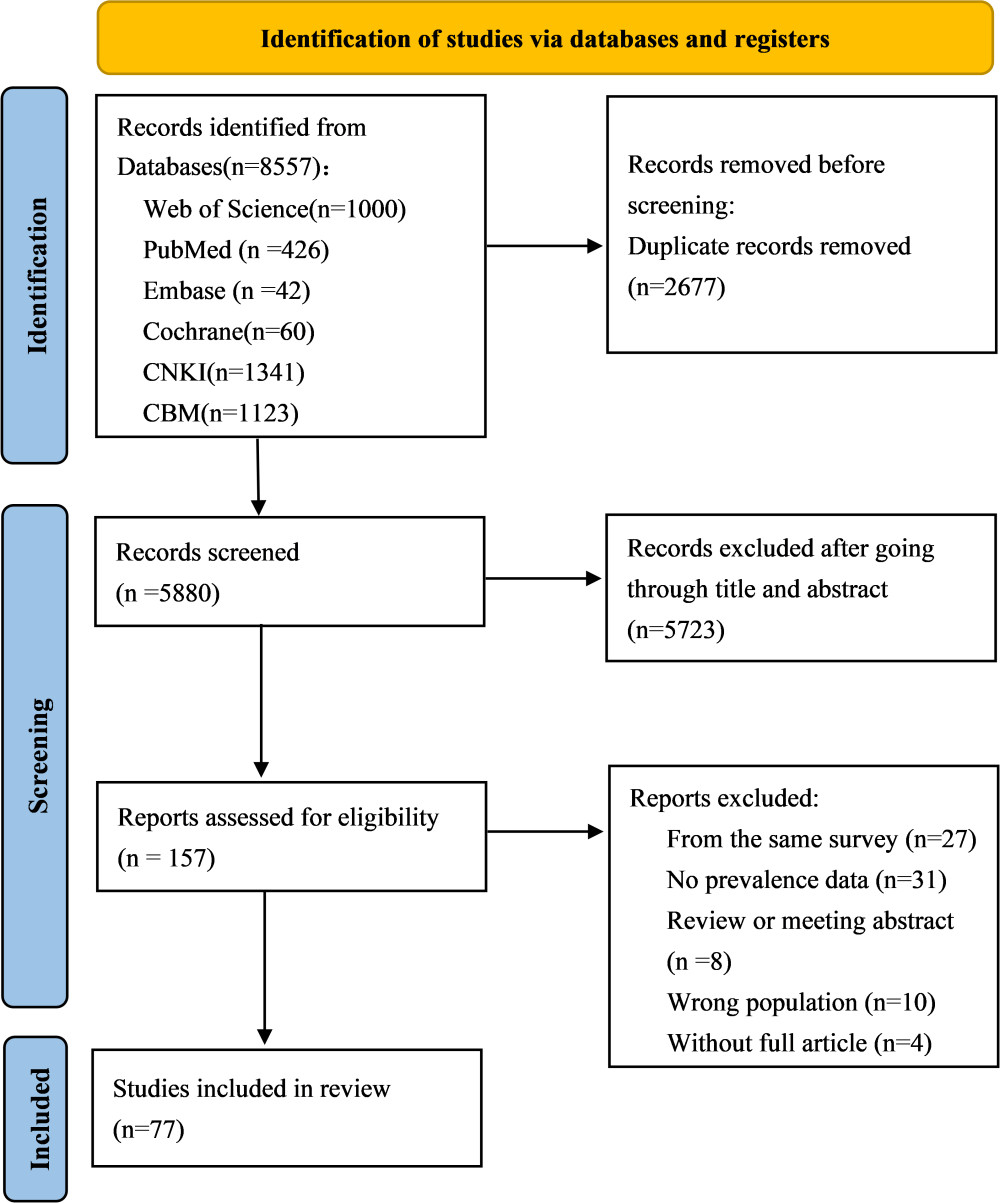
Flow chart of study selection.

The initial search yielded 8557 citations by searching eight electronic databases. Using EndNote X9 software, 2677 duplicate records were eliminated, then 5727 irrelevant studies were excluded by reading the titles and abstracts, the full texts of 157 potentially relevant studies were further read, and 77 studies were finally identified as meeting our requirements based on the inclusion criteria (**Figure 1**).

### 3.2 Study characteristics and quality assessment

This review included a total of 77 studies involving 108,1788 participants. The sample size ranged from 84 to 18,645, with an average of 1,303. Most study participants were students (n=56). Of the 77 included studies, a minority (n=9) used validated prospective tools to diagnose PMS. The characteristics of all included studies are reported in **Supplementary Table 2**.

Among the 77 included studies, 13 were rated high quality (AHRQ scores≥8), while the remaining 64 demonstrated medium quality (AHRQ scores 4-7). The risk of bias in the inclusion of studies arose mainly from item 9 (failure to explain how missing data were handled in the analyses), item 7 (failure to state the reason for excluding any patients from the study) and 11(failure to describe the desired follow-up outcome and the proportion of incomplete or subsequently obtained information); Additionally, item 2 (failure to develop inclusion and exclusion criteria) and 8 (failure to describe how confounders were assessed and controlled for) also contributed to the low quality of some studies (see **Supplementary Table 3**).

### 3.3 Prevalence of PMS in China

**Figure 2:**
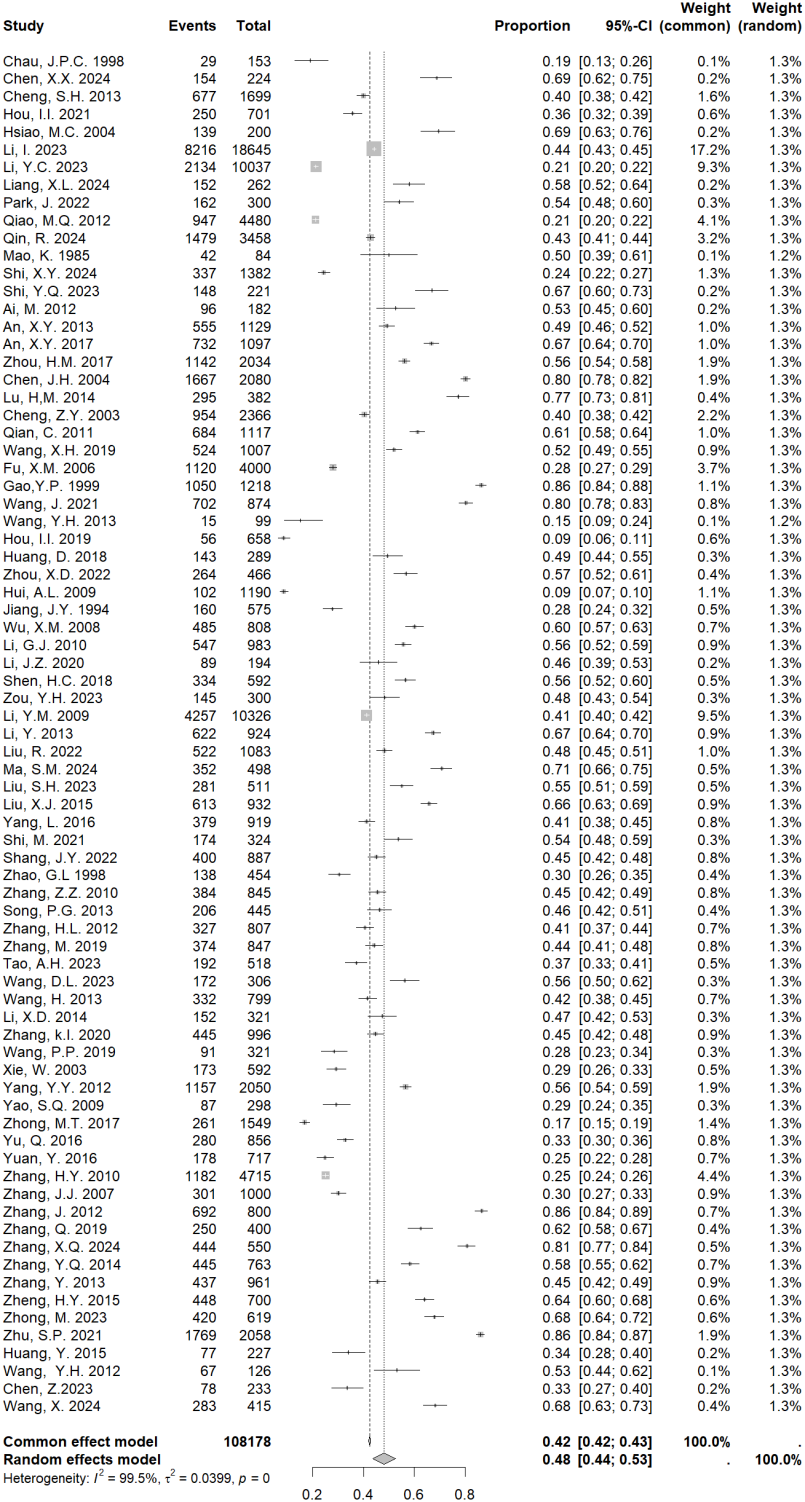
Forest plot of prevalence of PMS in China.

The heterogeneity test of the 77 included studies showed *I* ^2^ = 99.5%, *P*<0.001, Due to the obvious heterogeneity across studies, we used a random effects model in our meta-analyses. The results showed that the pooled prevalence of PMS in China was 48% [95% CI: 44-52%; **Figure 2**].

We investigated the statistical significance of factors considered to be possible sources of heterogeneity. Univariate meta-regression models were used to analyze variables including the use of validated prospective tools, region, screening tool, sample size, mean age, setting and study quality. As shown in **Table 1**, univariate meta-regression showed a significant effect of whether used prospective tools (with a lower prevalence of using prospective tools than not using it) (*P*=0.003). Whereas, there was no significant correlation between the prevalence of PMS and the study quality, region, sample size, setting, mean age and screening tool (**Supplementary Table 5**).

**Table 1:** Meta-regression analyses of the prevalence of PMS in China.

Variable	Categories	Beta-coefficient (95%CI)	*P*	*t* ^2^
Prospective tool	Yes	1.19[1.06-1.33]	0.003	0.020

### 3.4 Subgroup analyses

Significant heterogeneity was confirmed (*I* ^2^ = 99.5%, *P*<0.001). We performed subgroup analyses to identify factors that contributed to heterogeneity in the prevalence of PMS.

We found a maximum prevalence of 69% (95% CI: 55-82%) used the MDQ questionnaire, minimum for COPE with 25% (95% CI: 20-29%). The screening tool was further categorized into validated prospective and validated retrospective instrument, the pooled prevalence of PMS was 35% (95% CI: 27-42%) by using validated prospective instrument. For age group, the rate was lower among 15–20 age group than 20.1–25 age group, with 41% (95% CI:31-51%) versus 51% (95% CI:43-60%) (**Table 2**, **Supplementary Figures S1–3**).

**Table 2:** Subgroup analyses of the prevalence of PMS in China.

Subgroups	Categories	No. of studies	Prevalence (95%CI)	*I* ^2^ *(*%)	*P* values within subgroups	*P* values across subgroups
Screening tool	PMSS	29	0.50 [0.45-0.54]	97.9	<0.001	<0.001
	DRSP	6	0.33 [0.23-0.43]	98.4	<0.001	
	PSST	3	0.44 [0.09-0.80]	99.6	<0.001	
	COPE	3	0.25 [0.20-0.29]	91.1	<0.001	
	MDQ	3	0.69 [0.55-0.82]	97.9	<0.001	
	MHQ	4	0.46 [0.30-0.61]	98.6	<0.001	
	SPAF	3	0.46 [0.16-0.76]	99.7	<0.001	
Mean age	15-20	18	0.41 [0.31-0.51]	99.8	<0.001	<0.001
	20.1-25	17	0.51 [0.43-0.60]	99.3	<0.001	
	>25	8	0.51 [0.40-0.62]	98	<0.001	
Prospective tool	Yes	9	0.33 [0.26-0.40]	98.7	<0.001	<0.001
	No	39	0.51 [0.46-0.55]	99.2	<0.001	

Regarding the region, Northwestern China showed the highest prevalence of PMS 60% (95% CI: 47-92%), whereas North China showed the lowest 45% (95% CI: 36-54%). Among the included studies, 47 used validated tools and 30 did not. The pooled prevalence was 47% (95% CI:42-51%) for studies with validated tools, 50% (95% CI:43-58%) for those with non-validated tools. The prevalence of PMS in China varied by settings, with a higher estimate observed in hospital-based studies 57% (95% CI: 37-79%), followed by workplace settings 53% (95% CI: 43-59%). When the survey period was considered (pre-2010, 2010-2019, post-2020), the most recent period presented the highest prevalence with 58% (95% CI: 49-66%) in comparison with other two periods, with rates of 47% (95% CI: 33-62%) in pre-2010 and 51% (95% CI: 45-57%) in 2010-2019, respectively. The difference of pooled estimates between high and medium quality studies were different, the rate was higher among medium quality than high quality, with 49% (95% CI: 44-53%) versus 46% (95% CI: 38-53%) (**Supplementary Table 4**, **Supplementary Figures S4–8**).

Although we performed subgroup analyses to reduce the heterogeneity between the studies, high heterogeneity still exists as indicated by *I* ^2^(91.1% and above), which indicate the need to conduct the sensitivity test.

### 3.5 A leave-out-one sensitivity analyses

To examine heterogeneity, individual studies were excluded one by one to progressively determine the effect of each study on the overall prevalence of PMS. The results demonstrated that the values obtained were close to the overall prevalence, ranging from 48% to 49%. All values were within the estimated 95% CI (**Supplementary Figure 9**), indicating that excluding a single study did not significantly affect this meta - analyses.

### 3.6 Publication bias

**Figure 3:**
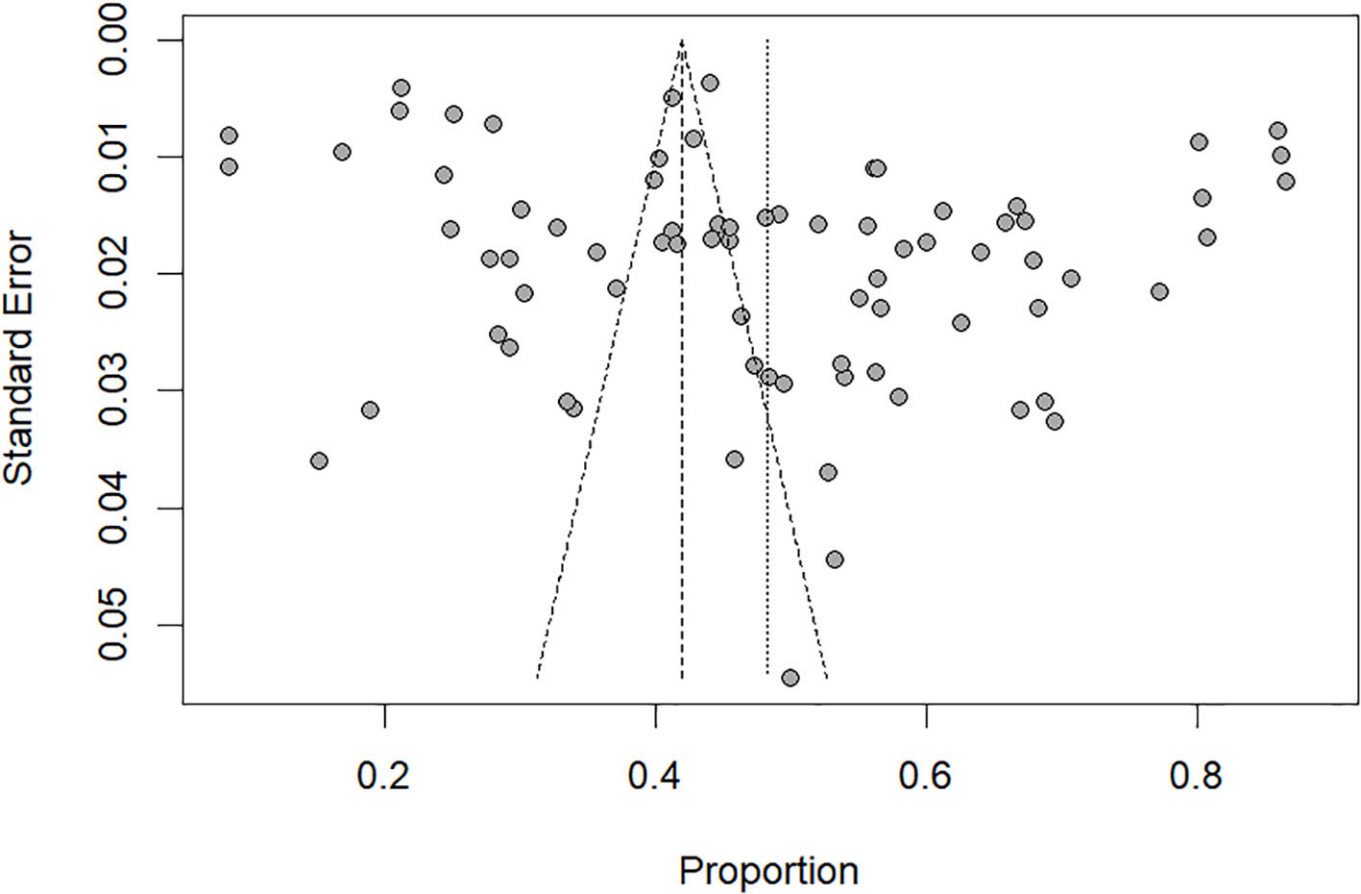
Funnel plot of publication bias on prevalence PMS inChina.

Visual inspection of the funnel plots (**Figure 3**) indicated a significant publication bias, which was also confirmed by Egger’s test (t=2.61, *P*=0.011).

## 4 Discussion

To our knowledge, this is the first meta-analyses exploring the prevalence of PMS in China. A total of 77 studies were included in this study, covering different regions and populations in China. The results indicated that the pooled prevalence of PMS in China was 48%. This result aligns with the conclusions of prior systematic review and meta-analyses conducted in India (35), Turkey (36), Ethiopia (37), Africa (38), and globally (39). However, it was significantly lower than the Iranian study (40), and variations in the populations can explain this difference studied, as well as their socio-cultural backgrounds and the screening tools used. That study involved only three validated questionnaires (DRSP, PSST, and PAS), whereas our review involved six.

The prevalence of PMS among Chinese women is high, which may be attributed to the following factors. First, from a socio-cultural perspective, previous studies have posited that PMS is a Western “culture-bound syndrome” (41, 42). However, accumulating epidemiological evidence from this study demonstrates that PMS represents a transcultural phenomenon with substantial prevalence among Chinese women. Indeed, influenced by traditional beliefs, although more women experience the discomfort associated with PMS, they consider discomfort during menstruation to be normal and choose to tolerate the pain. A study (43) found that among female college students, approximately one-third of restrained introverts opt to respond negatively and suffer in silence, thereby exacerbating their PMS distress, in contrast to the minority of extroverts who can promptly take steps to alleviate their distress. As indicated in the study (29), a greater propensity to experience inhibited emotions in daily life has been demonstrated to correlate with the manifestation of heightened premenstrual symptoms. Third, a sedentary lifestyle, imbalanced autonomic nervous system responses, poor sleep quality, and elevated occupational stress are also contributing factors (44–46). These factors may elevate the prevalence of PMS in China. While insufficient data on psychological and cultural factors preclude meta-analyses, we encouraged future studies to collect data on psychosocial variables (e.g., stress, stigma, personality traits) and include them as covariates in regression models to identify independent associations with PMS prevalence. Concurrently, prioritizing the psychosomatic health of Chinese women during the premenstrual phase is essential. Future research should focus on investigating PMS risk and protective factors to develop tailored preventive and interventional strategies.

In subgroup analyses, we found that the ratio of Chinese women classified as having PMS with the use of a non-validated tool was higher compared with studies using a validated tool, suggesting that screening instruments with the use of non-validated tool may overestimate the true prevalence. Significant variation in PMS prevalence emerged across screening tools. Prevalence estimates were substantially higher with MDQ (69%) than COPE (25%), possibly due to the MDQ utilizes retrospective self-reporting (33), COPE employ prospective symptom charts (23). This distinction enables prospective tools to provide more accurate prevalence estimates. In addition, the fact that current screening tools for PMS rely on foreign development. Consequently, the need to develop a screening tool for PMS applicable to Chinese women was emphasized in response to the significant differences caused by different tools. Further subdividing the validated tools into prospective and retrospective screening tools, we found that retrospective screening tools yielded prevalence estimates approximately 18% higher than prospective tools. Based on our findings and in alignment with diagnostic criteria (4, 47) which mandate prospective symptom documentation over two menstrual cycles for clinical diagnosis of PMS due to recall bias, we recommend adopting validated prospective instruments such as the DRSP and COPE for assessing PMS in Chinese population. While prospective screening tools are necessary to assess the presence or absence of disease and to obtain an accurate rate, it is worth noting that because they require the completion of two menstrual cycles to be documented, symptom ratings may not be completed for all participants (48). Patients who couldn’t complete their symptom diary may be affected by the severity of their symptoms, lack of motivation. Based on this, future research could aim to develop a diagnostic method that both provides a reliable diagnosis and reduces the burden on participants.

Our study also revealed that age may influence the prevalence of PMS among Chinese women. Notably, the prevalence of PMS increased with age, contrary to the results of the Iranian study (40), but aligns with the conclusions of Ji et al. (15). A potential explanation for our findings is that reproductive-age women may experience heightened economic, social, occupational, and marital pressures, thereby increasing their susceptibility to PMS (14). Strikingly, we observed a progressive increase in PMS prevalence over the study period, with a marked acceleration post-2020 (58%). With the rapid socioeconomic development of China, women’s social roles and expectations have changed, resulting in elevated levels of stress. This increased stress may affect the incidence and symptoms of PMS (49), as evidenced by established positive correlations between psychological stress and PMS manifestation (50). The COVID-19 pandemic has also adversely affected women’s menstruation. Women with COVID-19 infection have more severe symptoms of PMS than before, such as fatigue, headache, and irritability (51). Considering the above factors, future research should seek stress management interventions and mental health support. Furthermore, enhancing the work environment and increasing social support may reduce stress and anxiety among women.

Prevalence estimates of PMS varied significantly by study setting. Hospital-based studies reported a substantially higher prevalence (57%) compared to community-based studies (35%). This elevated prevalence in hospital settings is likely attributable to the inclusion of patients experiencing more severe symptoms due to underlying conditions (52). Furthermore, the frequent lack of reported sampling methods in hospital-based studies within our review suggests that selection bias also contribute to the observed difference. The reported prevalence of PMS among Chinese females in educational setting is consistent with findings from previous Africa studies (38). Subgroup analyses revealed marginally lower prevalence in high quality studies (46%) compared to medium quality studies (49%). The minimal difference (3%) suggests limited influence of study quality on pooled prevalence, supporting the reliability of our findings.

It has been noted that the prevalence and severity of PMS symptoms vary across countries and regions (14). Our review revealed a higher prevalence of PMS in Northwestern China (60%) compared to other regions. This disparity potentially reflects the region’s lower socioeconomic development relative to East China. Research indicates that lower socioeconomic status may elevate PMS risk by limiting individuals’ capacity to manage menstruation-related issues and access healthcare (53). Given this elevated prevalence (60%) in Northwestern China, targeted mental health interventions should be prioritized in this region. futhermore, current epidemiological studies on PMS in China predominantly focus on single provinces/cities within East and urban areas. Underrepresented regions (Northwestern, Northeastern, Central China) and rural populations remain inadequately studied. Future research should prioritize investigating the epidemiological characteristics of PMS among childbearing-age women in these underrepresented regions and rural areas. Key efforts should include comparative analyses of urban-rural prevalence patterns and identification of factors influencing these disparities.

### 4.1 Limitations

There are some limitations to our review. First, although subgroup analyses were performed, there was still a relatively high degree of heterogeneity among the studies reviewed, as it is often difficult to avoid heterogeneity in epidemiologic studies (54). Second, despite the measures we took to prevent missing publications (searching for articles in both Chinese and English databases), there was still significant publication bias, which must be considered when interpreting the results. Third, the included studies were conducted in urban areas, meaning our results cannot be generalized to the whole of China. Fourth, our study concentrated on the prevalence of PMS in China and did not address the relationship between risk factors and PMS. Therefore, this relationship must be taken into account when interpreting the findings. In order to further elucidate the potential modifying factors of PMS, further studies are required. Fifth and finally, bias was observed in instances because of incomplete information. For example, ethnic composition, income status, and age at menarche were understudied, thus preventing meta-analyses on the relevant outcomes. Future research is encouraged to explore these trends in Chinese women’s PMS.

## 5 Conclusion

We found a high prevalence of PMS was identified among Chinese women. To mitigate the negative impact of PMS on the health status of women of childbearing age, policies and guidelines, as well as effective preventive and treatment measures, should be developed for this population group to improve their health status.
