# Supplementary material for: The prevalence of premenstrual syndrome in China: a systematic review and meta-analyses
Source: Front Psychiatry. 2025 Sep 11;16:1640781. doi: 10.3389/fpsyt.2025.1640781 (PMC12461857; doi:10.3389/fpsyt.2025.1640781)
Supplement: Supplementary file 1 [file Supplementaryfile1.docx]

**Supplementary Material**

**The Prevalence of premenstrual syndrome in China: A systematic review and meta-analyses**

**Table of Contents**

| PRISMA checklist |
| --- |
| Supplementary Table 1 – Search strategy used in the current systematic review and meta-analyses |
| Supplementary Table 2 - Characteristics of included studies |
| Supplementary Table 3 - quality assessment of included studies |
| Supplementary Table 4 - Subgroup analyses of the prevalence of PMS in China |
| Supplementary Table 5 - Meta-regression analyses of the prevalence of PMS in China |
| Supplementary Figures 1- 8 subgroup analyses of the prevalence of PMS in China |
| Supplementary Figures 9- Sensitivity analyses of prevalence the PMS in Chinese |
| References |

| **Section and Topic** | **Item #** | **Checklist item** | **Location where item is reported** |
| --- | --- | --- | --- |
| **TITLE** | | |  |
| Title | 1 | Identify the report as a systematic review. | 1 |
| **ABSTRACT** | | |  |
| Abstract | 2 | See the PRISMA 2020 for Abstracts checklist. | 1 |
| **INTRODUCTION** | | |  |
| Rationale | 3 | Describe the rationale for the review in the context of existing knowledge. | 1 |
| Objectives | 4 | Provide an explicit statement of the objective(s) or question(s) the review addresses. | 1-2 |
| **METHODS** | | |  |
| Eligibility criteria | 5 | Specify the inclusion and exclusion criteria for the review and how studies were grouped for the syntheses. | 2 |
| Information sources | 6 | Specify all databases, registers, websites, organisations, reference lists and other sources searched or consulted to identify studies. Specify the date when each source was last searched or consulted. | 2 |
| Search strategy | 7 | Present the full search strategies for all databases, registers and websites, including any filters and limits used. | Supplementary Table 1 |
| Selection process | 8 | Specify the methods used to decide whether a study met the inclusion criteria of the review, including how many reviewers screened each record and each report retrieved, whether they worked independently, and if applicable, details of automation tools used in the process. | 2 |
| Data collection process | 9 | Specify the methods used to collect data from reports, including how many reviewers collected data from each report, whether they worked independently, any processes for obtaining or confirming data from study investigators, and if applicable, details of automation tools used in the process. | 2-3 |
| Data items | 10a | List and define all outcomes for which data were sought. Specify whether all results that were compatible with each outcome domain in each study were sought (e.g. for all measures, time points, analyses), and if not, the methods used to decide which results to collect. | 3 |
|  | 10b | List and define all other variables for which data were sought (e.g. participant and intervention characteristics, funding sources). Describe any assumptions made about any missing or unclear information. | 3 |
| Study risk of bias assessment | 11 | Specify the methods used to assess risk of bias in the included studies, including details of the tool(s) used, how many reviewers assessed each study and whether they worked independently, and if applicable, details of automation tools used in the process. | 2-3 |
| Effect measures | 12 | Specify for each outcome the effect measure(s) (e.g. risk ratio, mean difference) used in the synthesis or presentation of results. | 3 |
| Synthesis methods | 13a | Describe the processes used to decide which studies were eligible for each synthesis (e.g. tabulating the study intervention characteristics and comparing against the planned groups for each synthesis (item #5)). | 3 |
|  | 13b | Describe any methods required to prepare the data for presentation or synthesis, such as handling of missing summary statistics, or data conversions. | 3 |
|  | 13c | Describe any methods used to tabulate or visually display results of individual studies and syntheses. | 3 |
|  | 13d | Describe any methods used to synthesize results and provide a rationale for the choice(s). If meta-analysis was performed, describe the model(s), method(s) to identify the presence and extent of statistical heterogeneity, and software package(s) used. | 3 |
|  | 13e | Describe any methods used to explore possible causes of heterogeneity among study results (e.g. subgroup analysis, meta-regression). | 3 |
|  | 13f | Describe any sensitivity analyses conducted to assess robustness of the synthesized results. | 3 |
| Reporting bias assessment | 14 | Describe any methods used to assess risk of bias due to missing results in a synthesis (arising from reporting biases). | 3 |
| Certainty assessment | 15 | Describe any methods used to assess certainty (or confidence) in the body of evidence for an outcome. | 3 |
| **RESULTS** | | |  |
| Study selection | 16a | Describe the results of the search and selection process, from the number of records identified in the search to the number of studies included in the review, ideally using a flow diagram. | Figure 1 |
|  | 16b | Cite studies that might appear to meet the inclusion criteria, but which were excluded, and explain why they were excluded. | Figure 1 |
| Study characteristics | 17 | Cite each included study and present its characteristics. | Supplementary Table 2 |
| Risk of bias in studies | 18 | Present assessments of risk of bias for each included study. | Supplementary Table 3 |
| Results of individual studies | 19 | For all outcomes, present, for each study: (a) summary statistics for each group (where appropriate) and (b) an effect estimate and its precision (e.g. confidence/credible interval), ideally using structured tables or plots. | Figure 2 |
| Results of syntheses | 20a | For each synthesis, briefly summarise the characteristics and risk of bias among contributing studies. | Supplementary Table 2 |
|  | 20b | Present results of all statistical syntheses conducted. If meta-analysis was done, present for each the summary estimate and its precision (e.g. confidence/credible interval) and measures of statistical heterogeneity. If comparing groups, describe the direction of the effect. | Table 2  Supplementary Table 4 |
|  | 20c | Present results of all investigations of possible causes of heterogeneity among study results. | 7-8 |
|  | 20d | Present results of all sensitivity analyses conducted to assess the robustness of the synthesized results. | 11 |
| Reporting biases | 21 | Present assessments of risk of bias due to missing results (arising from reporting biases) for each synthesis assessed. | 11 |
| Certainty of evidence | 22 | Present assessments of certainty (or confidence) in the body of evidence for each outcome assessed. | 9 |
| **DISCUSSION** | | |  |
| Discussion | 23a | Provide a general interpretation of the results in the context of other evidence. | 12-15 |
|  | 23b | Discuss any limitations of the evidence included in the review. | 16 |
|  | 23c | Discuss any limitations of the review processes used. | 16 |
|  | 23d | Discuss implications of the results for practice, policy, and future research. | 15 |
| **OTHER INFORMATION** | | |  |
| Registration and protocol | 24a | Provide registration information for the review, including register name and registration number, or state that the review was not registered. | 4 |
|  | 24b | Indicate where the review protocol can be accessed, or state that a protocol was not prepared. | 4 |
|  | 24c | Describe and explain any amendments to information provided at registration or in the protocol. | NA |
| Support | 25 | Describe sources of financial or non-financial support for the review, and the role of the funders or sponsors in the review. | 17 |
| Competing interests | 26 | Declare any competing interests of review authors. | NA |
| Availability of data, code and other materials | 27 | Report which of the following are publicly available and where they can be found: template data collection forms; data extracted from included studies; data used for all analyses; analytic code; any other materials used in the review. | Supplementary |

**Supplementary Table 1 Search strategies of each database**

| Database | Index and Keyword terms |
| --- | --- |
| PubMed | (("Premenstrual Syndrome"[Mesh]) OR (((((((((Premenstrual Syndrome*[Title/Abstract]) OR (Premenstrual Dysphoric Disorder[Title/Abstract])) OR (Late Luteal Phase Dysphoric Disorder[Title/Abstract])) OR (Syndrome*, Premenstrual[Title/Abstract])) OR (Premenstrual Tension*[Title/Abstract])) OR (Tension*, Premenstrual[Title/Abstract])) OR (PMS[Title/Abstract])) OR (PMDD[Title/Abstract])) OR (LLPDD[Title/Abstract]))) AND (("China"[Mesh]) OR (((((China[Title/Abstract]) OR (Chinese[Title/Abstract])) OR (Hong Kong[Title/Abstract])) OR (mainland China[Title/Abstract])) OR (Macau'[Title/Abstract] OR (Taiwan[Title/Abstract]))) |
| Web of Science | TOPIC:(Premenstrual Syndrome* OR Syndrome*, Premenstrual OR Premenstrual Dysphoric Disorder OR Premenstrual Tension* OR Tension*, Premenstrual OR Late Luteal Phase Dysphoric Disorder) AND TOPIC: (China OR Chinese OR Beijing OR Hong Kong* OR Taiwan OR Mainland China OR Macau) |
| Embase | ('premenstrual syndrome'/exp OR 'premenstrual syndrome' OR 'premenstrual stress' OR 'premenstrual tension' OR 'premenstrual tension syndrome' OR 'syndrome*, premenstrual' OR 'premenstrual dysphoric disorder' OR 'late luteal phase dysphoric disorder' OR 'premenstrual tension*') AND ('China/exp OR 'China' OR 'Chinese' OR 'Beijing' OR 'Mainland China' OR 'Taiwan' OR 'Hong Kong' OR 'Macau') |
| Cochrane Library | MeSH descriptor: [Premenstrual Syndrome] explode all trees OR (premenstrual syndrome):ti,ab,kw OR (Premenstrual Dysphoric Disorder):ti,ab,kw OR (Late Luteal Phase Dysphoric Disorder):ti,ab,kw OR (Syndrome*, Premenstrua):ti,ab,kw OR (Premenstrual Tension*):ti,ab,kw OR (Tension*, Premenstrual):ti,ab,kw AND MeSH descriptor: [China] explode all trees OR (People's Republic of China):ti,ab,kw OR (Chinese):ti,ab,kw OR (Beijing):ti,ab,kw OR (hongkong):ti,ab,kw OR ("Taiwan"):ti,ab,kw OR (mainland China):ti,ab,kw OR (Macau):ti,ab,kw |
| CNKI | (主题: '经前期综合征' + '经前期焦虑障碍' + '经前期紧张' + 经前期综合症状+ '经前期紧张综合征') |
| WAN FANG | 主题:(经前期综合征) OR 主题:(经前期焦虑障碍) OR 主题:(经前期紧张) OR 主题:(经前期综合症状) OR 主题:(经前期紧张焦虑) OR 主题:(经前期综合症状) |
| VIP | [((((((题名或关键词=经前期综合征 OR 题名或关键词=经前综合征) OR 题名或关键词=经前期紧张综合症) OR 题名或关键词=经前综合症) OR 题名或关键词=经前期综合症) OR 题名或关键词=经前焦虑障碍) OR 题名或关键词=经前期紧张焦虑)](https://qikan.cqvip.com/Qikan/search/index?LngMySearHistoryIdGuid=0e557245-dcb1-461e-99e6-6525f457447f&from=Qikan_Article_History) |
| CBM | ("经前期综合征"[不加权:扩展] OR "经前期综合征" OR "经前期焦虑障碍" OR "经前期紧张" OR "经前期综合症状") |

**Supplementary Table 2 Characteristics of included studies**

| **Study** | **Study period** | **Provence** | **Setting** | **Sample size** | **PMS (%)** | **Diagnostic tool** | **Mean age (±SD)** | **Quality score** |
| --- | --- | --- | --- | --- | --- | --- | --- | --- |
| Chau, J.P.C. 1998 | NR | Hong Kong | school | 153 | 29(19) | MSQ | 15.21 | 6 |
| Chen, X.X. 2024 | 2022-2023 | Guang Dong | workplace | 224 | 154(68.7) | MDQ | 34.00±6.05 | 7 |
| Cheng, S.H. 2013 | NR | Tai Wan | school | 1699 | 677(39.85) | PMSS | 21.58±4.00 | 8 |
| Hou, l.l. 2021 | NR | Jiang Su | school | 701 | 250(35.7) | PMSS | 19.72±3.14 | 8 |
| Hsiao, M.C. 2004 | 2000-2001 | Tai Wan | hospital | 200 | 139(69.5) | C | NR | 7 |
| Chen, Z.2023 | 2019-2020 | Shan Dong | school | 233 | 78(33.5) | DRSP | NR | 7 |
| Wang, X. 2024 | 2023 | Jiang Su | workplace | 415 | 283(68.2) | PMSS | NR | 7 |
| Li, l. 2023 | 2020-2022 | Shan Dong | workplace | 18645 | 8216(44.1) | PMSS | 33.50±5.89 | 9 |
| Li, Y.C. 2023 | 2019 | Si Chuan | school | 10037 | 2134(21.3) | COPE | 14.63±1.63 | 9 |
| Liang, X.L. 2024 | 2018-2020 | Guang Dong | hospital | 262 | 151(57.6) | PSST | NR | 9 |
| Park, J. 2022 | 2019 | Hong Kong | school | 300 | 162(54) | SPAF | NR | 7 |
| Qiao, M.Q. 2012 | NR | * Mainland China | community | 4480 | 947(21.1) | DRSP | NR | 8 |
| Qin, R. 2024 | 2020 | * Mainland China | school | 3458 | 1479(42.8) | PMSS | 19.48±1.45 | 7 |
| Shi, X.Y. 2024 | 2021-2023 | Si Chuan | school | 1382 | 337(24.4) | COPE | 20.0±1.41 | 6 |

| **Study** | **Study period** | **Provence** | **Setting** | | **Sample size** | | **PMS (%)** | **Diagnostic tool** | **Mean age (±SD)** | **Quality score** |
| --- | --- | --- | --- | --- | --- | --- | --- | --- | --- | --- |
| Shi, Y.Q. 2023 | 2021-2022 | Shang Hai | school | | 221 | | 148(67) | PSST | 21.69±2.50 | 9 |
| Ai, M. 2012 | 2011 | Jiang Su | school | | 182 | | 96(52,7) | MHQ | NR | 6 |
| An, X.Y. 2013 | 2012 | Gui Zhou | school | | 1129 | | 555(49.2) | PMSS | 19.8±1.5 | 5 |
| An, X.Y. 2017 | 2014 | Gui Zhou | school | | 1097 | | 732(66.7) | PMSS | 20.65±3.07 | 7 |
| Zhou, H.M. 2017 | 2015 | Si Chuan | school | | 2034 | | 1142(56.1) | A | 19.5±1.1 | 6 |
| Chen, J.H. 2004 | NR | Zhe Jiang | school | | 2080 | | 1667(80.1) | Q | NR | 5 |
| Lu, H.M. 2014 | 2007-2014 | Hu Bei | hospital | | 382 | | 295(77.22) | D | 37±3.11 | 6 |
| Cheng, Z.Y. 2003 | 2002 | Nei MengGu | school | | 2366 | | 954(44.74) | MHQ | NR | 6 |
| Qian, C. 2011 | NR | Zhe Jiang | school | | 1117 | | 684(44.74) | Q | 19.74±0.74 | 6 |
| Wang, X.H. 2019 | 2017-2018 | Liao Ning | workplace | | 1007 | | 524(52) | PMSS | 33.2±1.6 | 8 |
| Fu, X.M. 2006 | 2005 | Guang Dong | hospital | | 4000 | | 1120(28) | Q | 23.3±4.5 | 5 |
| Gao, Y.P. 1999 | 1997 | He Nan | school | | 1218 | | 1050(86.2) | Q | NR | 6 |
| Wang, J. 2021 | 2019 | Shan Xi | school | | 874 | | 702(80.2) | MDQ | 18.9±1.35 | 7 |
| Wang, Y.H. 2013 | NR | Fu Jian | school | | 99 | | 15(15.15) | DRSP | 20.82±1.15 | 7 |
| **Study** | **Study period** | **Provence** | | **Setting** | | **Sample size** | **PMS (%)** | **Diagnostic tool** | **Mean age (±SD)** | **Quality score** |
| Huang, D. 2018 | NR | Si Chuan | | school | | 289 | 143(49.5) | PMSS | 19.9±1.4 | 6 |
| Zhou, X.D. 2022 | NR | Xin Jiang | | school | | 466 | 264(56.7) | PMSS | NR | 8 |
| Hui, A.L. 2009 | NR | He Nan | | school | | 1190 | 102(8.6) | Q | 19.8 | 5 |
| Jiang, J.Y. 1994 | NR | Shang Hai | | school | | 575 | 160(27.8) | MHQ | NR | 5 |
| Wu, X.M. 2008 | NR | Guang Dong | | workplace | | 808 | 485(60.02) | PMSS | NR | 6 |
| Li, G.J. 2010 | 2007 | Shan Xi | | school | | 983 | 547(55.65) | Q | NR | 6 |
| Li, J.Z. 2020 | 2018-2019 | An Hui | | school | | 194 | 89(45.87) | PMSS | 20.53±0.88 | 7 |
| Shen, H.C. 2018 | 2017 | An Hui | | school | | 592 | 334(56.4) | MDQ | 20.38 | 7 |
| Zou, Y.H. 2023 | 2021 | Liao Ning | | community | | 300 | 145(48) | PMSS | 31.14±9.89 | 8 |
| Li, Y.M. 2009 | 2008 | * Mainland China | | school | | 10326 | 4257(41.2) | Q | NR | 6 |
| Li, Y. 2013 | 2013 | Guang Dong | | school | | 924 | 622(67.32) | Q | 21.3±1.4 | 7 |
| Liu, R. 2022 | 2021-2022 | An Hui | | school | | 1083 | 522(67.32) | PMSS | 19.285±1.358 | 7 |
| Ma, S.M. 2024 | 2023 | Tian Jin | | school | | 498 | 352(70.68) | PMSS | NR | 7 |
| Liu, S.H. 2023 | 2022 | * Mainland China | | school | | 511 | 281(54.99) | PMSS | NR | 7 |

| **Study** | **Study period** | **Provence** | **Setting** | **Sample size** | **PMS (%)** | **Diagnostic tool** | **Mean age (±SD)** | **Quality score** |
| --- | --- | --- | --- | --- | --- | --- | --- | --- |
| Yang, L. 2016 | NR | Shan Xi | school | 919 | 379(41.24) | DRSP | 21.97±1.384 | 6 |
| Shi, M. 2021 | 2019 | An Hui | school | 324 | 174(53.7) | PMSS | 19.89±0.86 | 8 |
| Shang, J.Y. 2022 | 2017 | Gui Zhou | school | 887 | 400(45.1) | PMSS | NR | 7 |
| Zhao, G.L 1998 | NR | Bei Jing | Mixed | 454 | 138(30.4) | PMSS | 19.5±1.1 | 5 |
| Zhang, Z.Z. 2010 | NR | Shan Dong | school | 845 | 384(45.4) | PMSS | NR | 7 |
| Song, P.G. 2013 | 2011 | Bei Jing | school | 445 | 206(46.3) | A | 30.6±8.96 | 7 |
| Zhang, H.L. 2012 | 2010-2011 | Ao Men | community | 807 | 327(40.5) | A | 20.0±1.66 | 5 |
| Zhang, M. 2019 | 2017-2018 | An Hui | school | 847 | 374(44.2) | PMSS | 21.2±1.7 | 8 |
| Tao, A.H. 2023 | 2022 | Zhe Jiang | school | 518 | 192(37.1) | PMSS | NR | 7 |
| Wang, D.L. 2023 | 2022 | Ning Xia | workplace | 306 | 172(56,21) | PMSS | NR | 8 |
| Wang, H. 2013 | 2009-2012 | Jiang Su | school | 799 | 332(41.55) | DRSP | 29.72±7.00 | 6 |
| Li, X.D. 2014 | NR | Bei Jing | workplace | 321 | 152(47.4) | DRSP | NR | 6 |
| Zhang, k. l. 2020 | NR | Gui Zhou | school | 996 | 445(44.7) | PMSS | 19.24±1.49 | 5 |
| Xie, W. 2003 | 2002-2003 | Shang Hai | school | 592 | 173(31.59) | COPE | NR | 6 |

| **Study** | **Study period** | **Provence** | **Setting** | **Sample size** | **PMS (%)** | **Diagnostic tool** | **Mean age (±SD)** | **Quality score** |
| --- | --- | --- | --- | --- | --- | --- | --- | --- |
| Yao, S.Q. 2009 | NR | He Bei | workplace | 298 | 87(29.19) | NR | 19.78±1.51 | 7 |
| Zhong, M.T. 2017 | NR | Hu Nan | school | 1549 | 261(16.8) | SPAF | 19.97±1.56 | 6 |
| Yu, Q. 2016 | 2015-2016 | Liao Ning | school | 856 | 280(32.7) | PMSS | 20.6±1.46 | 6 |
| Yuan, Y. 2016 | 2014 | Fu Jian | school | 717 | 178(24.82) | Q | NR | 6 |
| Zhang, H.Y. 2010 | NR | * Mainland China | Mixed | 4715 | 1182(25.1) | Q | NR | 5 |
| Zhang, J.J. 2007 | 2006 | Shan Dong | community | 1000 | 301(30.28) | Q | NR | 6 |
| Zhang, J. 2012 | NR | Fu Jian | school | 800 | 692(86.52) | Q | NR | 5 |
| Zhang, Q. 2019 | NR | Liao Ning | school | 400 | 250(62.5) | PMSS | 21.04±1.59 | 5 |
| Zhang, X.Q. 2024 | 2023 | Jiang Su | school | 550 | 444(80.73) | PMSS | NR | 6 |
| Zhang, Y.Q. 2014 | 2012 | Guang Xi | school | 763 | 445(58.32) | Q | 20.7±1.4 | 6 |
| Zhang, Y. 2013 | NR | Zhe Jiang | school | 961 | 437(45.5) | PMSS | 21.37±1.41 | 6 |
| Zheng, H.Y. 2015 | NR | Gan Su | school | 700 | 448(64) | MHQ | 20.25±1.44 | 6 |
| Zhong, M. 2023 | 2023 | Guang Dong | school | 619 | 420(67.85) | SPAF | 15.25±3.14 | 6 |
| Zhu, S.P. 2021 | 2020 | Guang Dong | school | 2058 | 1769(85.96) | Q | 20.7±1.4 | 5 |

| **Study** | **Study period** | **Provence** | **Setting** | **Sample size** | **PMS (%)** | **Diagnostic tool** | **Mean age (±SD)** | **Quality score** |
| --- | --- | --- | --- | --- | --- | --- | --- | --- |
| Hou, l. l. 2019 | NR | Jiang Su | school | 658 | 56(8.5) | PSST | 19.56±3.16 | 5 |
| Liu, X.J. 2015 | 2014 | Shan Dong | school | 932 | 613(65.77) | Q | 21.4±1.3 | 7 |
| Wang, P.P. 2019 | NR | Shan Dong | school | 321 | 91(28.3) | Q | 19.24±1.49 | 5 |
| Yang, Y.Y. 2012 | 2011 | Fu Jian | school | 2050 | 1157(56.4) | Q | NR | 5 |
| Mao, K. 1985 | NR | Hong Kong | workplace | 84 | 42(50) | Q | NR | 5 |
| Huang, Y. 2015 | NR | Bei Jing | NR | 227 | 77(33.9) | PMSS | NR | 7 |
| Wang, Y.H. 2012 | NR | Bei Jing | hospital | 126 | 67(53.2) | PMSD | NR | 6 |

Note: Q=Questionnaire designed by the researcher; A=Questionnaire based on ACOG; C=Checklist based on ICD-10;D=Questionnaire based on DSM-Ⅳ; NR=Not Reported; MSQ=Menstrual Symptom Questionnaire; MDQ=Menstrual Distress Questionnaire; PSST=Premenstrual Symptoms Screening Tool; SPAF=Shortened Premenstrual Assessment Form; MHQ=Menstrual Health Questionnaire; COPE=Calendar of Premenstrual Experiences; DRSP=Daily Record of Severity of Problems; PMSS= Premenstrual Syndrome Scale PMSD=Premenstrual Syndrome Diary *Mainland China: Refers to the 31 provincial-level administrative divisions under the jurisdiction of the People's Republic of China, excluding the Special Administrative Regions of Hong Kong and Macao, and the province of Taiwan.

**Supplementary Table 3** Methodological quality appraisal results based on the AHRQ tool for included studies

| Study | 1 | 2 | 3 | 4 | 5 | 6 | 7 | 8 | 9 | 10 | 11 | Total Score | quality |
| --- | --- | --- | --- | --- | --- | --- | --- | --- | --- | --- | --- | --- | --- |
| Chau, J.P. 1998 | Y | N | N | Y | Y | Y | U | Y | U | Y | U | 6 | M |
| Chen, X.X. 2024 | Y | Y | Y | Y | Y | Y | U | N | U | Y | N | 7 | M |
| Cheng, S.H. 2013 | Y | N | Y | Y | Y | Y | Y | Y | U | Y | N | 8 | H |
| Hou, l. l. 2021 | Y | Y | N | Y | Y | Y | Y | Y | U | Y | N | 8 | H |
| Hsiao, M.C. 2004 | Y | Y | Y | Y | Y | Y | N | N | N | N | Y | 7 | M |
| Huang, Y. 2015 | Y | Y | N | Y | Y | Y | Y | N | N | Y | N | 7 | M |
| Wang, Y.H. 2012 | Y | Y | N | Y | Y | Y | Y | Y | N | N | N | 7 | M |
| Li, l. 2023 | Y | Y | Y | Y | Y | Y | Y | Y | N | N | N | 9 | H |
| Li, Y.C. 2023 | Y | Y | Y | Y | Y | Y | Y | Y | N | Y | N | 9 | H |
| Liang, X.L. 2024 | Y | Y | Y | Y | Y | Y | Y | Y | N | Y | N | 9 | H |
| Park, J. 2022 | Y | Y | Y | Y | Y | Y | NA | Y | NA | N | N | 7 | M |
| Qiao, M.Q. 2012 | Y | Y | N | Y | Y | Y | Y | N | N | Y | Y | 8 | H |
| Qin, R. 2024 | Y | N | Y | Y | Y | Y | Y | Y | N | N | N | 7 | M |
| Shi, X.Y. 2024 | Y | N | Y | Y | Y | Y | N | Y | N | N | N | 6 | M |
| Mao, K. 1985 | Y | N | N | Y | Y | Y | U | N | N | Y | N | 5 | M |
| Shi, Y.Q. 2023 | Y | Y | Y | Y | Y | Y | Y | Y | N | Y | N | 9 | H |
| Ai, M. 2012 | Y | N | Y | Y | Y | Y | N | N | N | Y | N | 6 | M |
| An, X.Y. 2013 | Y | N | Y | Y | U | Y | N | N | N | Y | N | 5 | M |
| An, X.Y. 2017 | Y | N | Y | Y | Y | Y | N | Y | N | Y | N | 7 | M |
| Zhou, H.M. 2017 | Y | N | Y | Y | Y | Y | N | N | N | Y | N | 6 | M |
| Chen, J.H. 2004 | Y | N | N | Y | Y | Y | N | N | U | Y | N | 5 | M |
| Lu, H.M. 2014 | Y | N | Y | Y | Y | Y | N | N | U | Y | N | 6 | M |
| Cheng, Z.Y. 2003 | Y | N | Y | Y | Y | Y | N | N | N | Y | N | 6 | M |
| Qian, C. 2011 | Y | N | N | Y | Y | Y | N | Y | N | Y | N | 6 | M |
| Wang, X.H. 2019 | Y | Y | Y | Y | Y | Y | N | Y | N | Y | N | 8 | H |

| Study | 1 | 2 | 3 | 4 | 5 | 6 | 7 | 8 | 9 | 10 | 11 | Total Score | quality |
| --- | --- | --- | --- | --- | --- | --- | --- | --- | --- | --- | --- | --- | --- |
| Fu, X.M. 2006 | Y | N | Y | Y | Y | Y | N | N | N | N | N | 5 | M |
| Gao, Y P. 1999 | Y | N | Y | Y | Y | Y | N | N | N | Y | N | 6 | M |
| Wang, J. 2021 | Y | Y | Y | Y | Y | Y | N | N | N | Y | N | 7 | M |
| Wang, Y.H. 2013 | Y | Y | N | Y | Y | Y | Y | N | N | Y | N | 7 | M |
| Hou, l. l. 2019 | Y | N | N | Y | Y | Y | N | N | N | Y | N | 5 | M |
| Huang, D. 2018 | Y | N | N | Y | Y | Y | N | Y | N | Y | N | 6 | M |
| Zhou, X.D. 2022 | Y | Y | Y | Y | Y | Y | N | Y | N | Y | N | 8 | H |
| Hui, A.L. 2009 | Y | N | N | Y | Y | Y | N | N | NA | Y | N | 5 | M |
| Jiang, J.Y. 1994 | Y | N | N | Y | Y | Y | N | N | N | Y | N | 5 | M |
| Wu, X.M. 2008 | Y | N | N | Y | Y | Y | N | Y | N | Y | N | 6 | M |
| Li, G.J. 2010 | Y | N | Y | Y | Y | Y | N | N | N | Y | N | 6 | M |
| Li, J.Z. 2020 | Y | Y | Y | Y | Y | Y | N | N | N | Y | N | 7 | M |
| Shen, H.C. 2018 | Y | Y | Y | Y | Y | Y | N | N | N | Y | N | 7 | M |
| Zou, Y.H. 2023 | Y | Y | Y | Y | Y | Y | N | Y | N | Y | N | 8 | H |
| Li, Y.M. 2009 | Y | N | Y | Y | Y | Y | N | Y | N | N | N | 6 | M |
| Li, Y. 2013 | Y | N | Y | Y | Y | Y | N | Y | N | Y | N | 7 | M |
| Liu, R. 2022 | Y | Y | Y | Y | Y | Y | N | N | N | Y | N | 7 | M |
| Ma, S.M. 2024 | Y | Y | Y | Y | Y | Y | N | N | N | Y | N | 7 | M |
| Liu, S.H. 2023 | Y | Y | Y | Y | Y | Y | N | N | N | Y | N | 7 | M |
| Liu, X.J. 2015 | Y | N | Y | Y | Y | Y | N | Y | N | Y | N | 7 | M |
| Yang, L. 2016 | Y | N | N | Y | Y | Y | N | Y | N | Y | N | 6 | M |
| Shi, M. 2021 | Y | Y | Y | Y | Y | Y | N | Y | N | Y | N | 8 | H |
| Shang, J.Y. 2022 | Y | Y | Y | Y | Y | Y | N | N | N | Y | N | 7 | M |
| Zhao, G.L 1998 | Y | N | N | Y | Y | Y | N | N | NA | Y | N | 5 | M |
| Zhang, Z.Z. 2010 | Y | N | N | Y | Y | Y | Y | Y | N | Y | N | 7 | M |

| Study | 1 | 2 | 3 | 4 | 5 | 6 | 7 | 8 | 9 | 10 | 11 | Total Score | quality |
| --- | --- | --- | --- | --- | --- | --- | --- | --- | --- | --- | --- | --- | --- |
| Song, P.G. 2013 | Y | N | Y | Y | Y | Y | Y | N | N | Y | N | 7 | M |
| Zhang, H.L. 2012 | Y | N | Y | Y | Y | Y | N | N | N | N | N | 5 | M |
| Zhang, M. 2019 | Y | Y | Y | Y | Y | Y | N | Y | N | Y | N | 8 | H |
| Tao, A.H. 2023 | Y | N | Y | Y | Y | Y | N | Y | N | Y | N | 7 | M |
| Wang, D.L. 2023 | Y | Y | Y | Y | Y | Y | N | Y | N | Y | N | 8 | H |
| Wang, H. 2013 | Y | N | Y | Y | Y | Y | N | N | N | Y | N | 6 | M |
| Li, X.D. 2014 | Y | Y | N | Y | Y | Y | N | N | N | Y | N | 6 | M |
| Zhang, k. l. 2020 | Y | N | N | Y | Y | Y | N | N | N | Y | N | 5 | M |
| Wang, P.P. 2019 | Y | N | N | Y | Y | Y | N | N | N | Y | N | 5 | M |
| Xie, W. 2003 | Y | N | Y | Y | Y | Y | N | N | N | Y | N | 6 | M |
| Yang, Y.Y. 2012 | Y | N | Y | Y | Y | Y | N | N | N | N | N | 5 | M |
| Yao, S.Q. 2009 | Y | Y | N | Y | Y | Y | N | Y | N | Y | N | 7 | M |
| Zhong, M.T. 2017 | Y | N | N | Y | Y | Y | N | Y | N | Y | N | 6 | M |
| Yu, Q. 2016 | Y | N | N | Y | Y | Y | N | Y | N | Y | N | 6 | M |
| Yuan, Y. 2016 | Y | N | Y | Y | Y | Y | N | N | N | Y | N | 6 | M |
| Zhang, H.Y. 2010 | Y | Y | N | Y | Y | Y | N | N | N | N | N | 5 | M |
| Zhang, J.J. 2007 | Y | Y | Y | Y | Y | Y | N | N | N | N | N | 6 | M |
| Zhang, J. 2012 | Y | N | N | Y | Y | Y | N | N | N | Y | N | 5 | M |
| Zhang, Q. 2019 | Y | Y | N | Y | Y | Y | N | N | N | N | N | 5 | M |
| Zhang, X.Q. 2024 | Y | N | Y | Y | Y | Y | N | N | N | Y | N | 6 | M |
| Zhang, Y.Q. 2014 | Y | N | Y | Y | Y | Y | N | N | N | Y | N | 6 | M |
| Zhang, Y. 2013 | Y | N | N | Y | Y | Y | N | Y | N | Y | N | 6 | M |
| Zheng, H.Y. 2015 | Y | N | Y | Y | Y | Y | N | N | N | Y | N | 6 | M |
| Zhong, M. 2023 | Y | N | Y | Y | Y | Y | N | N | N | Y | N | 6 | M |
| Zhu, S.P. 2021 | Y | N | Y | Y | Y | Y | N | N | N | N | N | 5 | M |
| Chen, Z.2023 | Y | Y | Y | Y | Y | Y | N | Y | N | N | N | 6 | M |
| Wang, X. 2024 | Y | Y | Y | Y | Y | Y | N | N | N | Y | N | 6 | M |

**Supplementary Table 4 Subgroup analyses of the prevalence of PMS in China**

| **Subgroups** | **Categories** | **No. of studies** | **Prevalence (95%CI)** | ***I*^2^(%)** | ***P* values within subgroups** | ***P* values across subgroups** |
| --- | --- | --- | --- | --- | --- | --- |
| Region | North China | 9 | 0.45[0.36-0.54] | 97.5 | <0.001 | 0.523 |
|  | East China | 30 | 0.48[0.41-0.55] | 99.5 | <0.001 |  |
|  | Southern China | 12 | 0.55[0.44-0.65] | 99.7 | <0.001 |  |
|  | Northwestern China | 5 | 0.60[0.47-0.72] | 98.9 | <0.001 |  |
|  | Northeastern China | 4 | 0.49[0.37-0.61] | 97.7 | <0.001 |  |
|  | Southwestern China | 8 | 0.46[0.35-0.57] | 99.7 | <0.001 |  |
|  | The central of China | 4 | 0.47[0.08-0.87] | 99.9 | <0.001 |  |
| Validated tools | Yes | 47 | 0.47[0.42-0.51] | 99.4 | <0.001 | 0.419 |
|  | No | 30 | 0.50[0.43-0.58] | 99.8 | <0.001 |  |
| Setting | School | 56 | 0.49[0.43-0.54] | 99.7 | <0.001 | 0.060 |
|  | Workplace | 9 | 0.53[0.45-0.61] | 97.4 | <0.001 |  |
|  | Community | 4 | 0.35[0.23-0.46] | 98.5 | <0.001 |  |
|  | Hospital | 5 | 0.57[0.40-0.74] | 99.4 | <0.001 |  |
| Study period | Pre-2010 | 8 | 0.47[0.33-0.62] | 99.7 | <0.001 | 0.358 |
|  | 2010-2019 | 21 | 0.51[0.45-0.57] | 99.6 | <0.001 |  |
|  | Post-2020 | 15 | 0.58[0.49-0.66] | 99.6 | <0.001 |  |
| Study quality | Medium | 64 | 0.49[0.44-0.53] | 99.6 | <0.001 | 0.524 |
|  | High | 13 | 0.46[0.38-0.53] | 99.6 | <0.001 |  |

**Supplementary Table 5 Meta-regression analyses of the prevalence of PMS in China**

| **Variable** | **Categories** | **Beta-coefficient(95%CI)** | ***P*** | ***t*^2^** |
| --- | --- | --- | --- | --- |
| Mean age | 15-20 | 0.91[0.77-1.07] | 0.244 | 0.035 |
|  | 20.1-25 | 1.00[0.85-1.19] | 0.930 |  |
| Setting | Community | 0.80[0.62-1.03] | 0.087 | 0.033 |
|  | School | 0.92[0.77-1.10] | 0.382 |  |
|  | Workplace | 0.96[0.78-1.19] | 0.725 |  |
| Screening tool | COPE | 0.81[0.51-0.81] | 0.082 | 0.019 |
|  | DRSP | 0.89[0.72-1.10] | 0.268 |  |
|  | MHQ | 1.00[0.80-1.25] | 0.994 |  |
|  | PMSS | 1.04[0.87-1.24] | 0.632 |  |
|  | PSST | 0.97[0.76-1.24] | 0.823 |  |
|  | MDQ | 1.26[0.99-1.60] | 0.058 |  |
| Region | East China | 1.00[0.82-1.24] | 0.928 | 0.035 |
|  | North China | 0.98[0.78-1.24] | 0.892 |  |
|  | Northeastern China | 1.02[0.78-1.33] | 0.885 |  |
|  | Northwestern China | 1.14[0.88-1.47] | 0.317 |  |
|  | Southern China | 1.09[0.87-1.36] | 0.452 |  |
|  | Southwestern China | 0.99[0.79-1.25] | 0.942 |  |
| Sample size | >2000 | 1.00[0.85-1.17] | 0.986 | 0.034 |
|  | 500-1000 | 1.03[0.90-1.18] | 0.649 |  |
|  | <500 | 1.05[0.91-1.21] | 0.492 |  |
| Study quality | Medium | 1.03[0.92-1.16] | 0.607 | 0.033 |


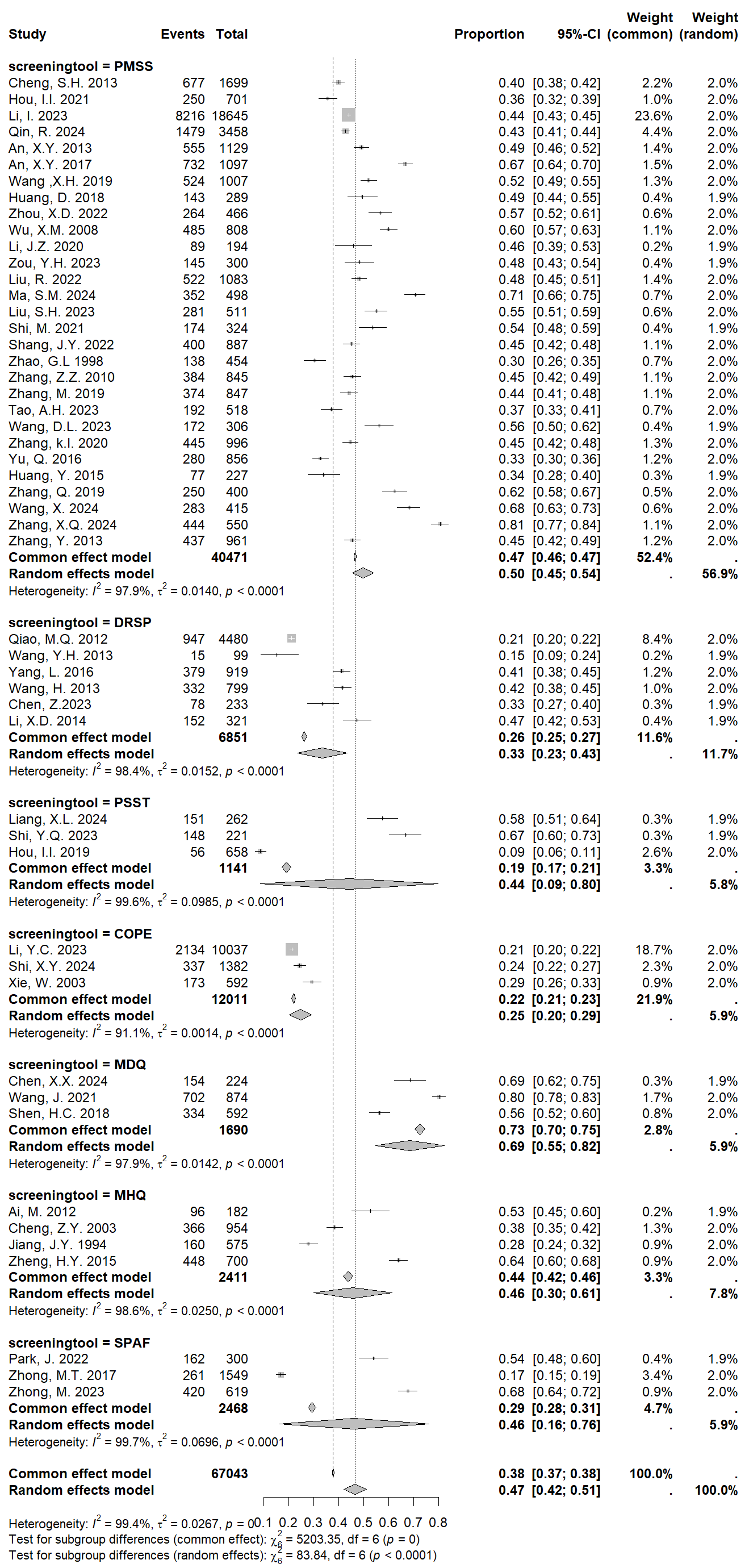


**Supplementary Figure 1** Forest plot of pooled prevalence, grouped by screening tool


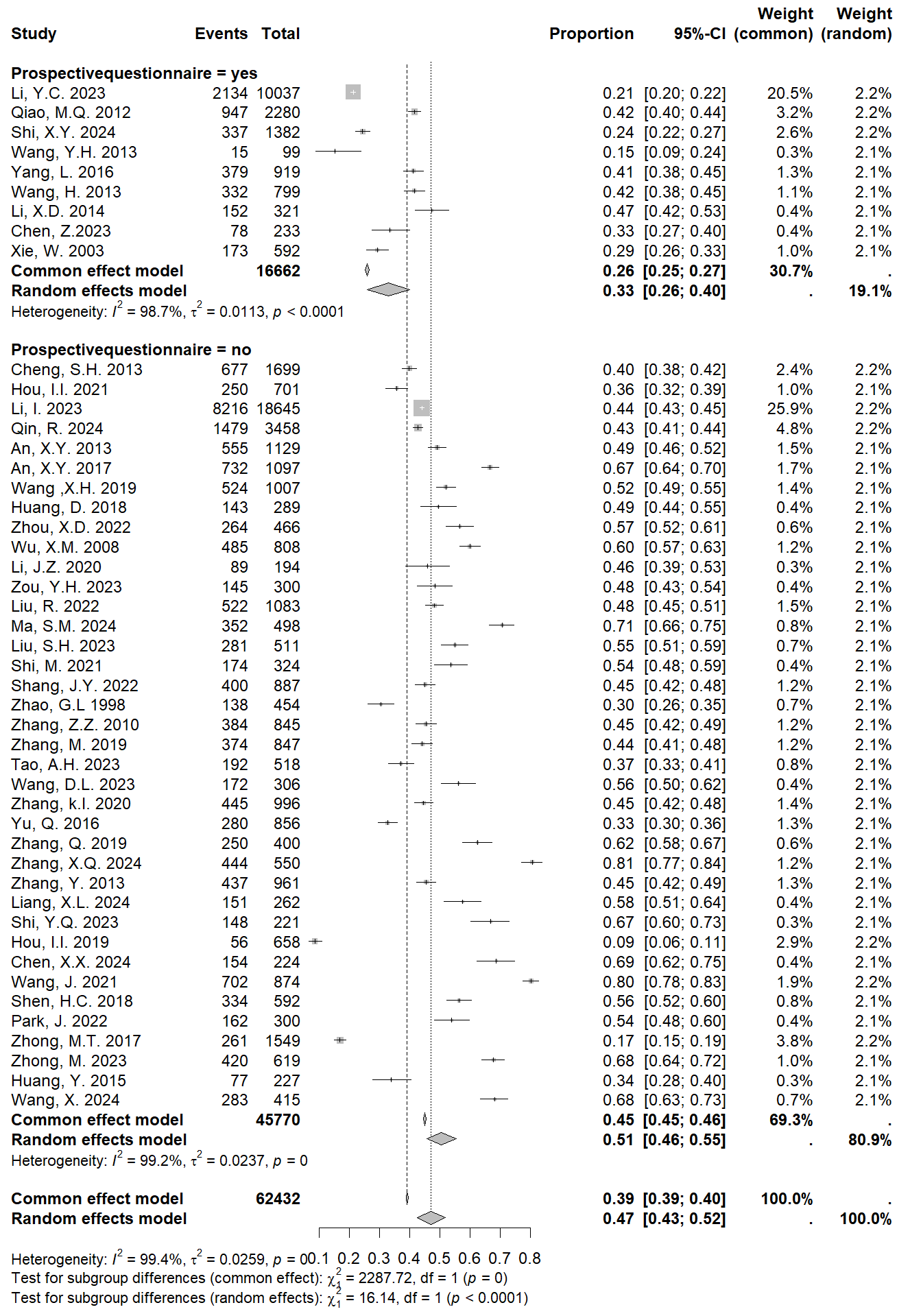


**Supplementary Figure 2** Forest plot of pooled prevalence, grouped by whether a prospective questionnaire was used


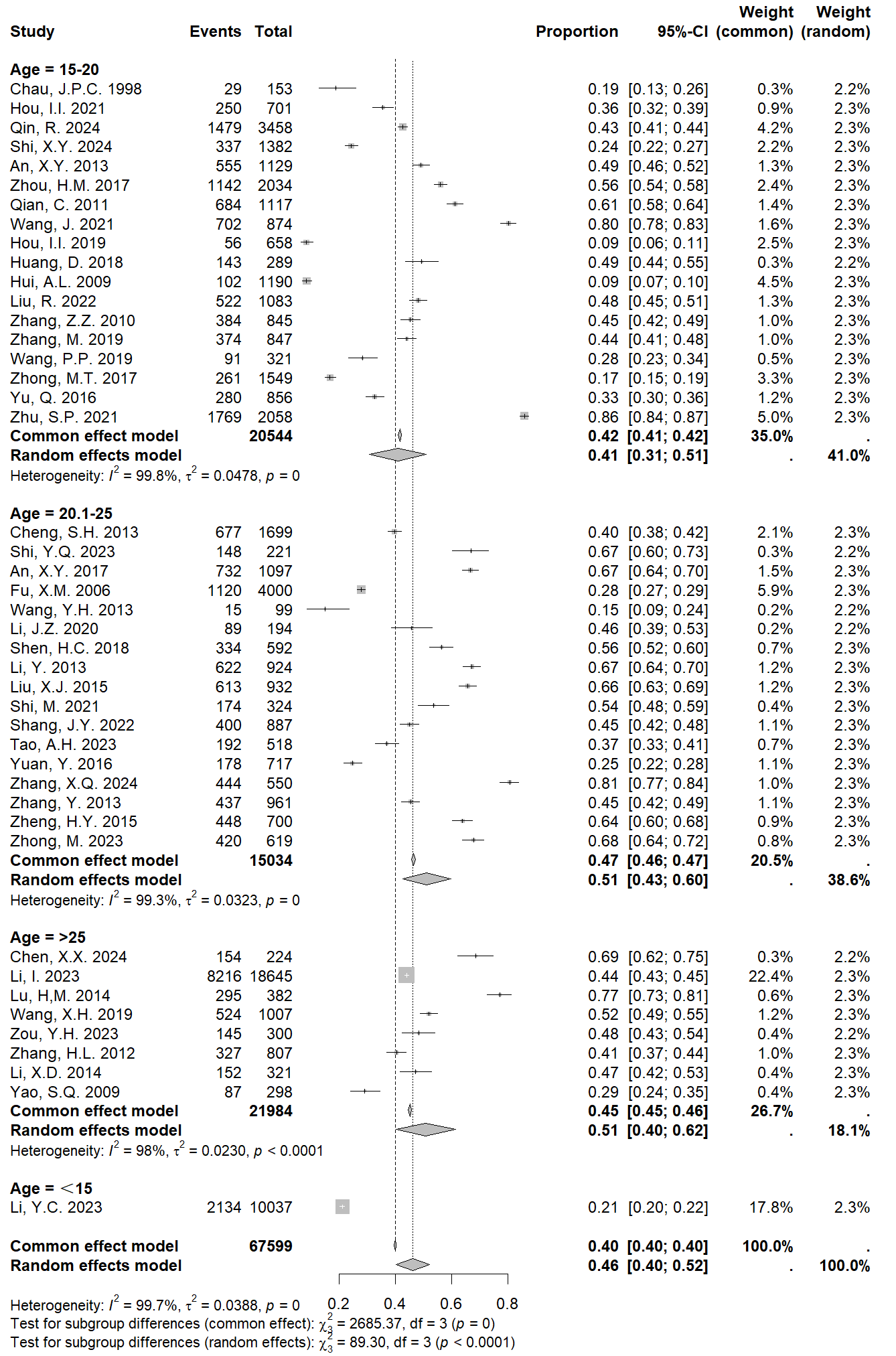


**Supplementary Figure 3** Forest plot of pooled prevalence, grouped by mean age


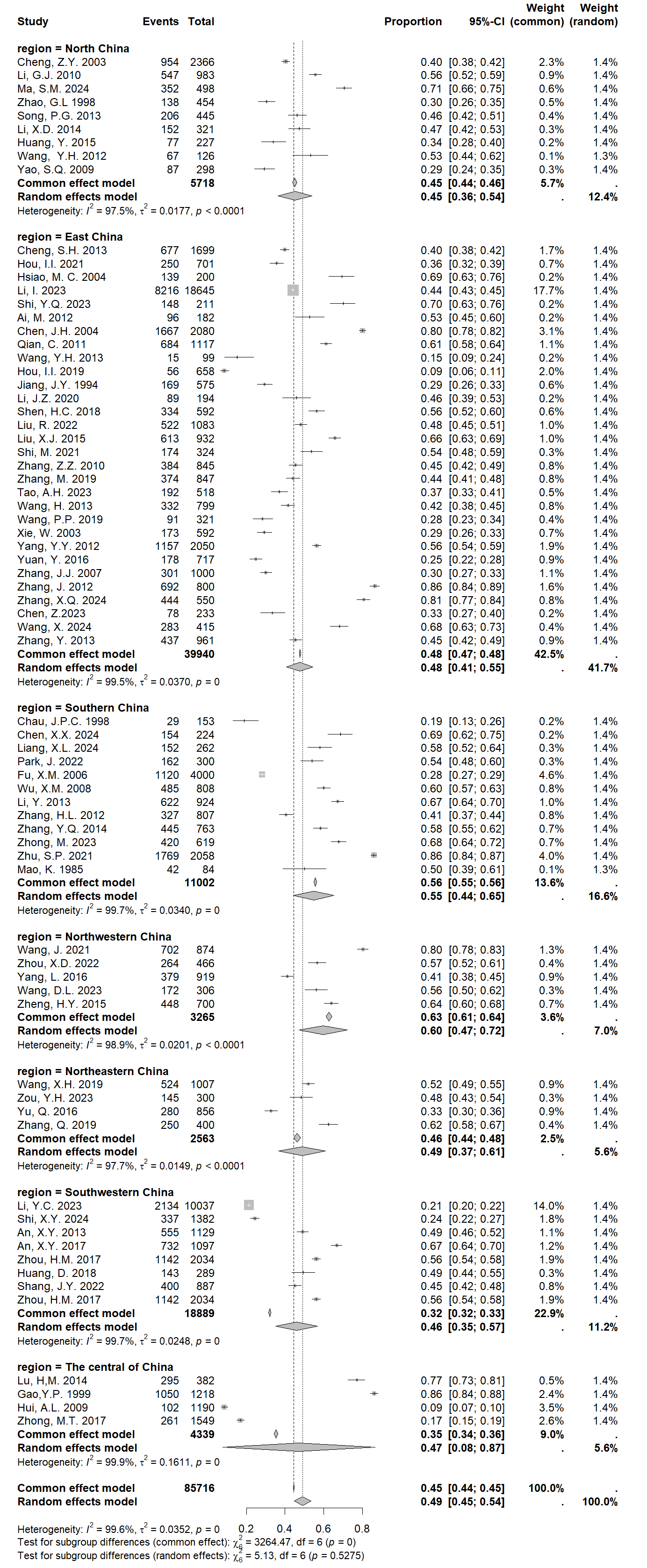


**Supplementary Figure 4** Forest plot of pooled prevalence, grouped by region


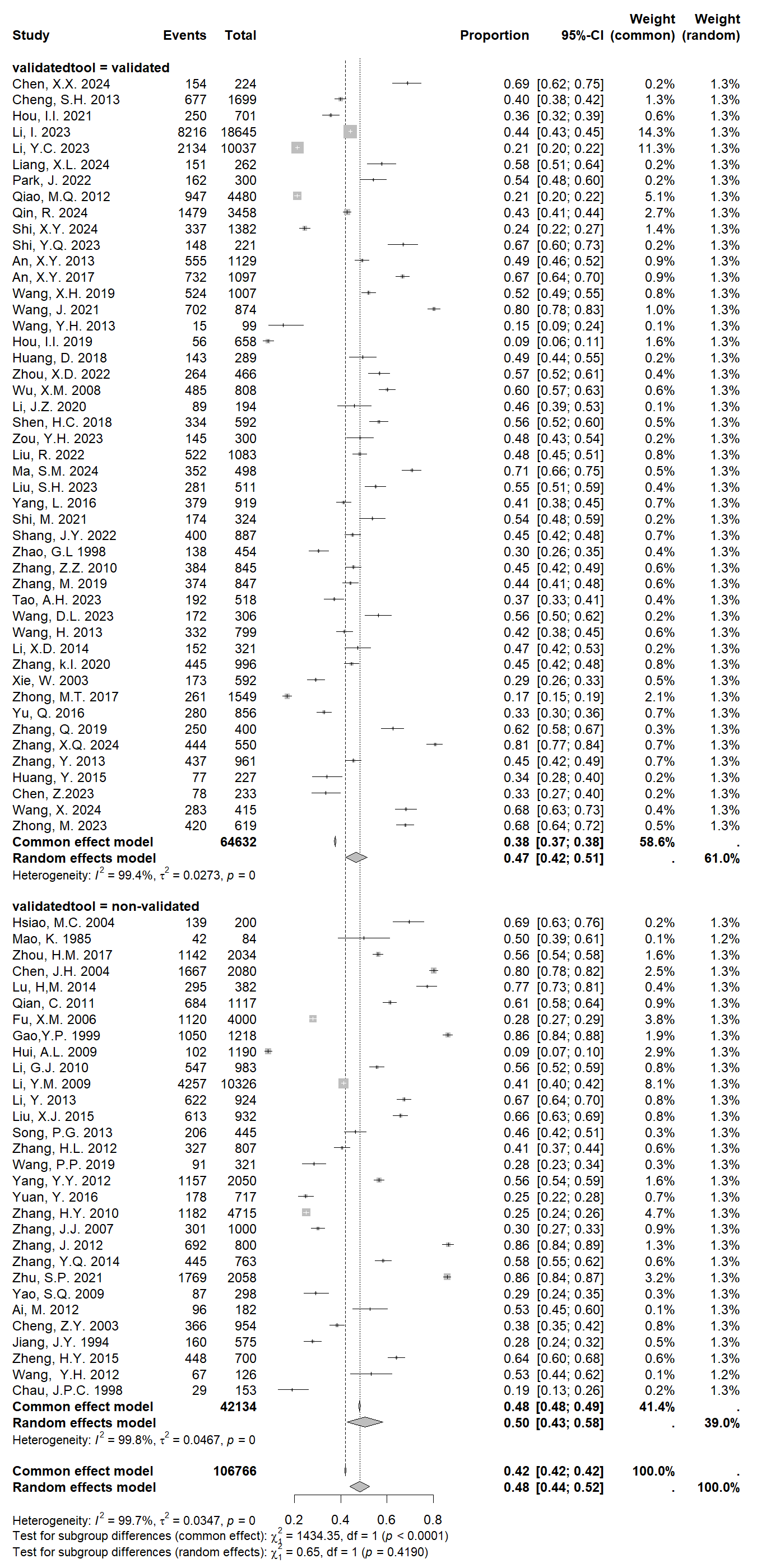


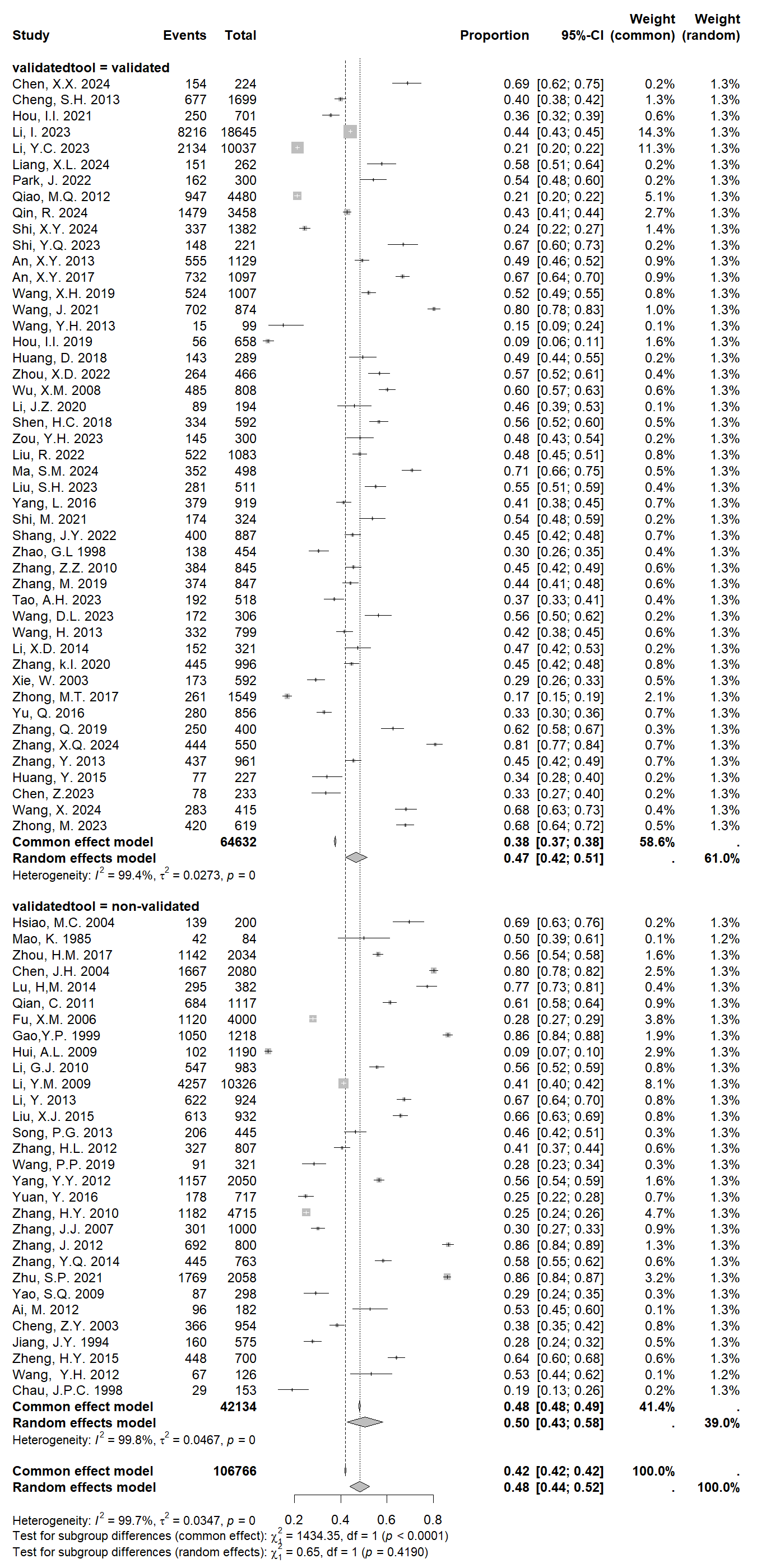


**Supplementary Figure 5** Forest plot of pooled prevalence, grouped by whether a validated tools was used


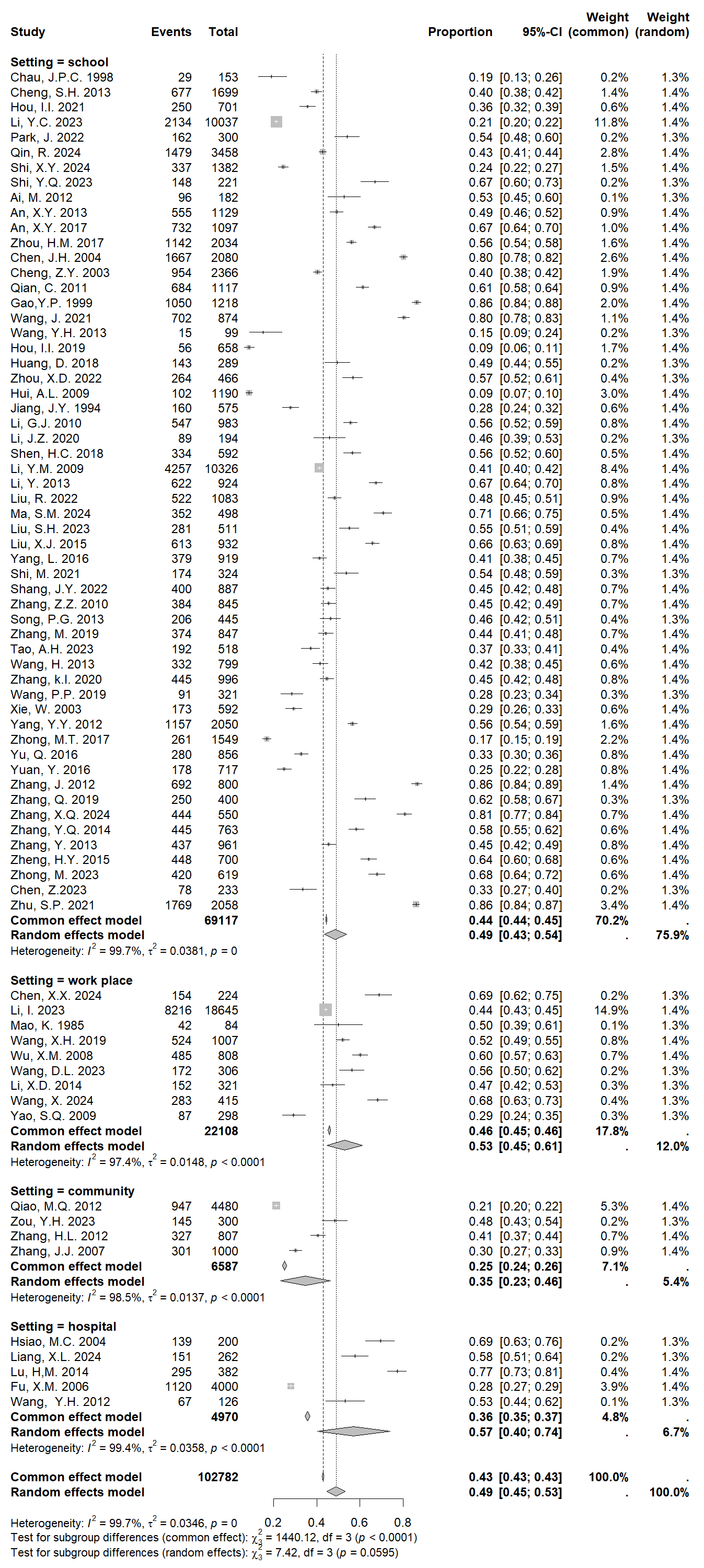


**Supplementary Figure 6** Forest plot of pooled prevalence, grouped by setting


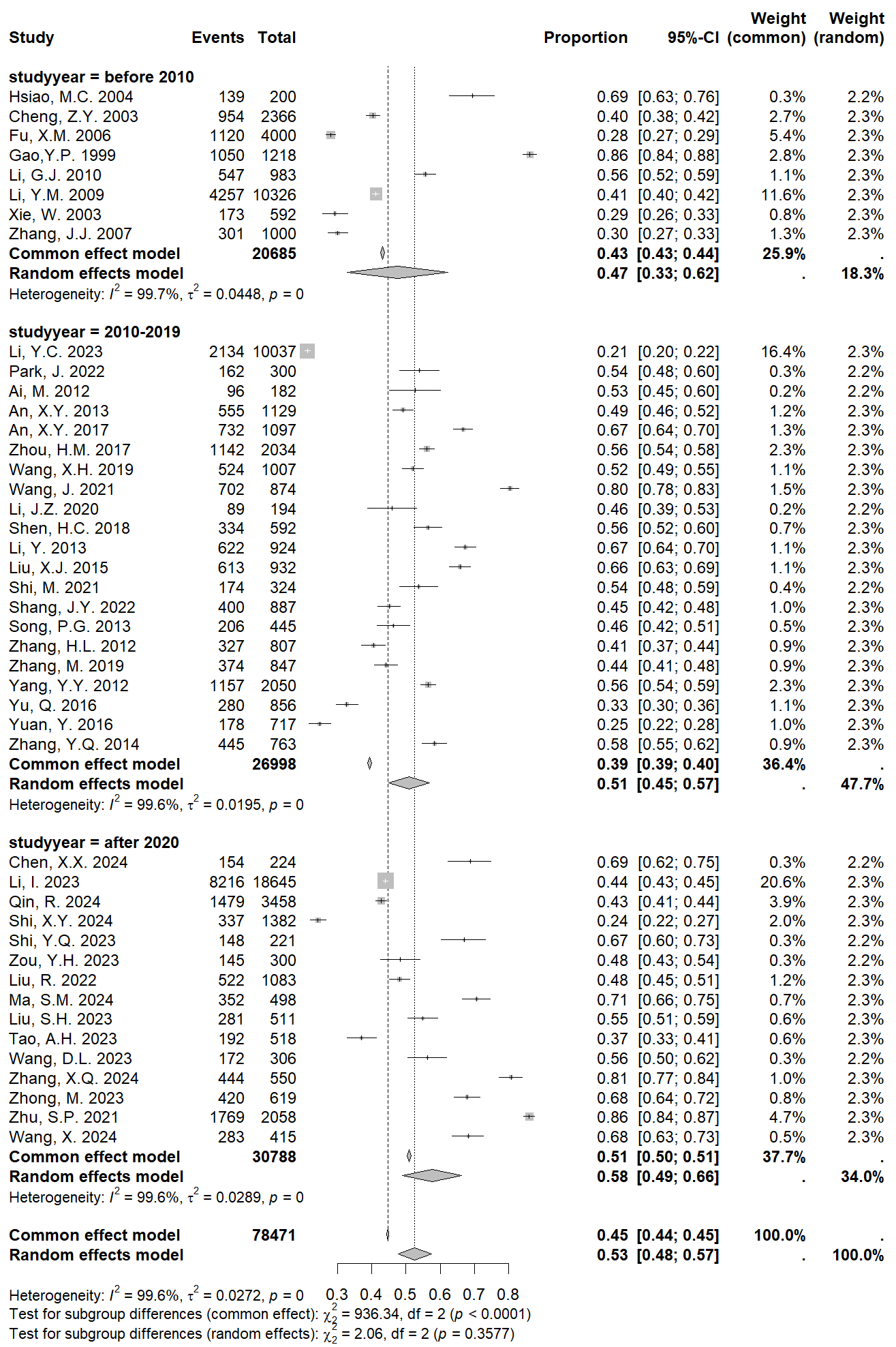


**Supplementary Figure 7** Forest plot of pooled prevalence, grouped by study year


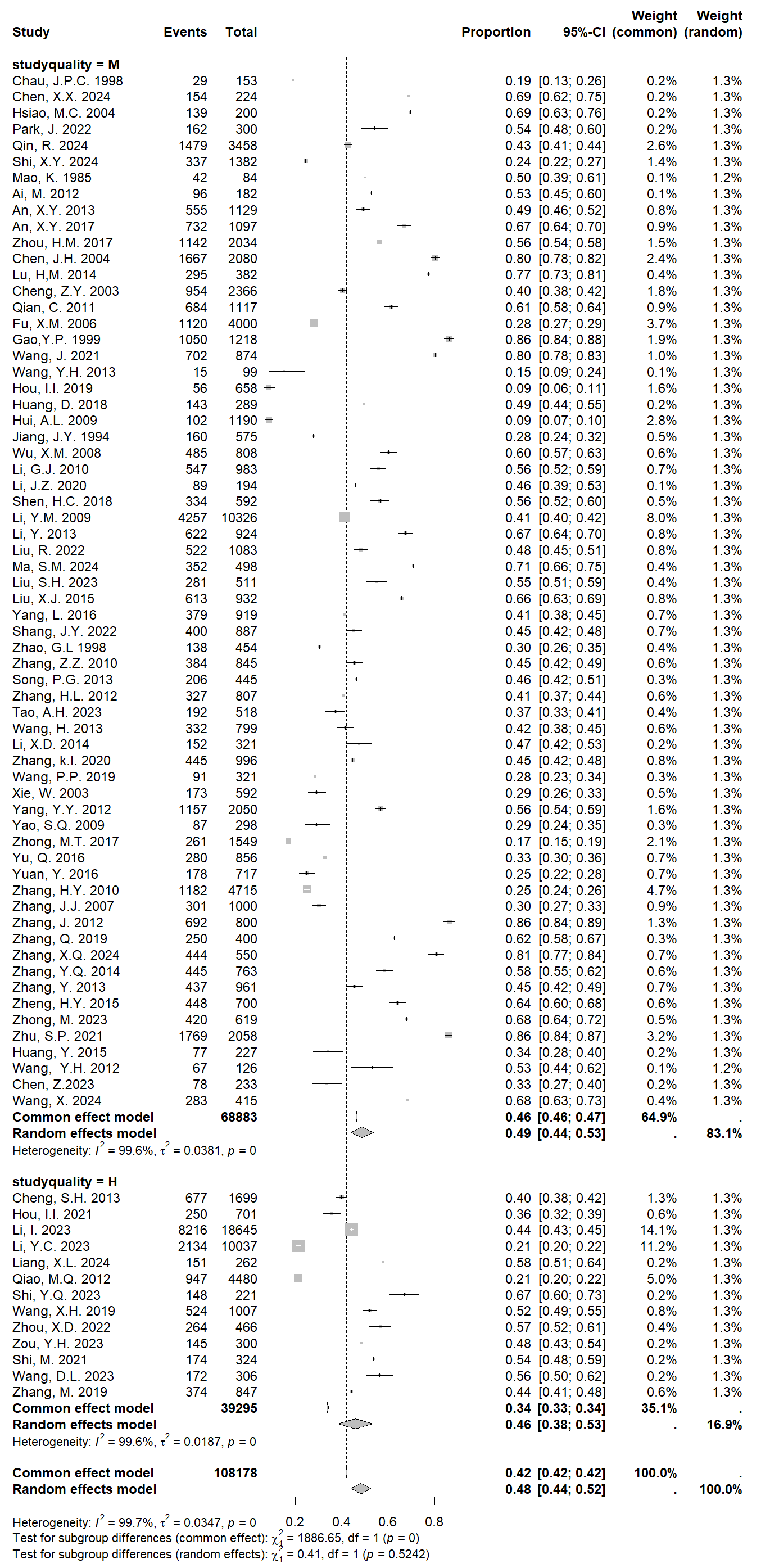


**Supplementary Figure 8** Forest plot of pooled prevalence, grouped by study quality


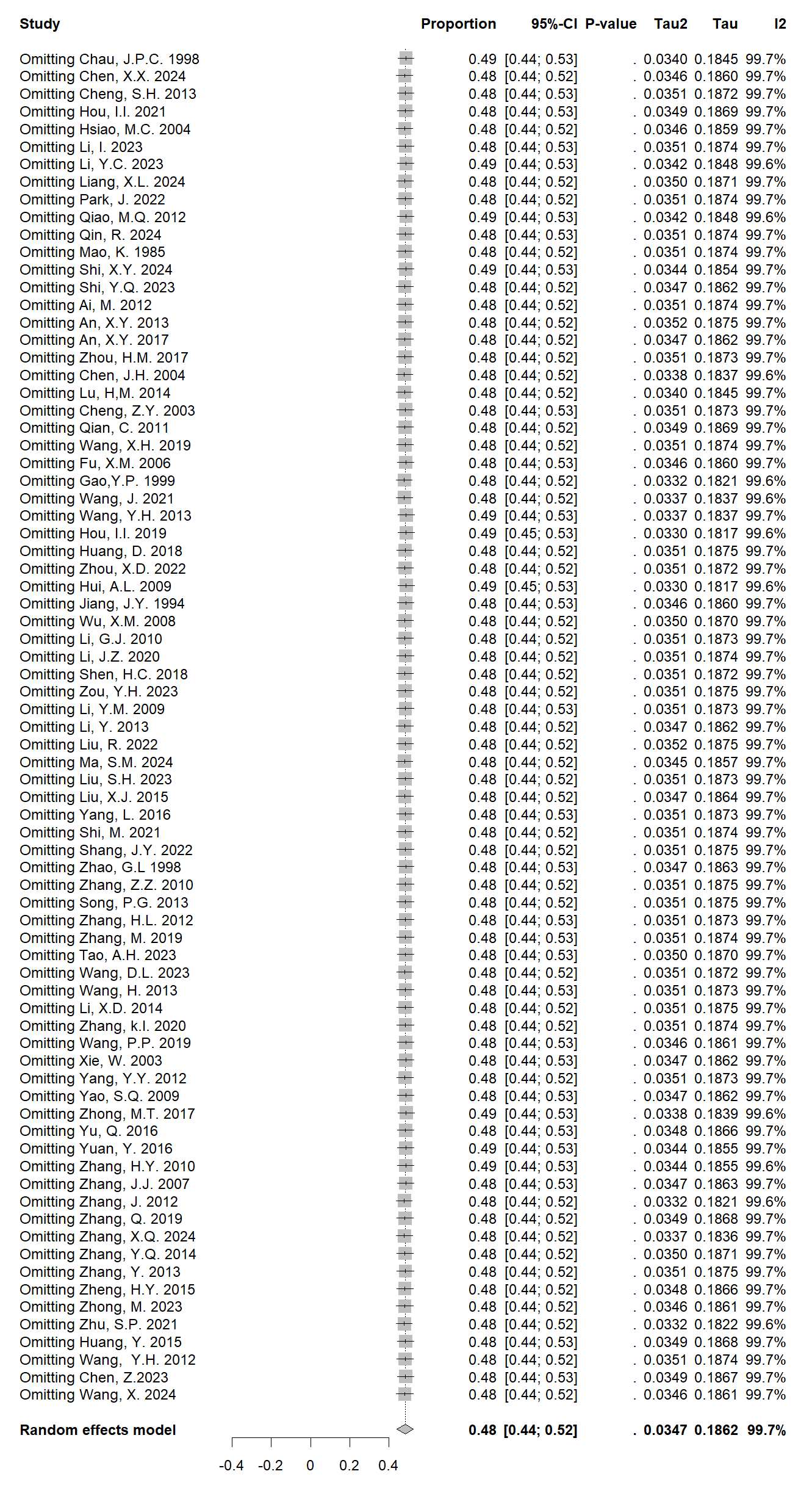


**Supplementary Figures 9** Sensitivity analyses of the prevalence of PMS in China

**References**

Ai, M., Xu, L.Y., Pan, Q., 2012. A survey of menstrual disorders, menstrual knowledge, attitudes, and behaviors of freshman female students in two majors at a higher education institution. J. Qiqihar Uni. Med. 33(20): 2807-8.

An, X.Y., Li, X.L., Huang, L., 2017. A study on the main influencing factors of premenstrual syndrome in female college students and psychological intervention measure. Psychol. Doc. 23(19): 316-7.

An, X.Y., Yu, Y.S., Yao, R., 2013. Relationship between premenstrual syndrome and type C personality in female medical college students. MCHC 28.

Chau, J.P.C., Chang, A.M., Chang, A.M.Z., 1998. Relationship between premenstrual tension syndrome and anxiety in Chinese adolescents. J. Adolesc. Health 22(3): 247-9.

Chen, J.H., 2004. The effect of premenstrual tension syndrome on examination performance in female students of Lishui Health College. Chin. J. Sch. Health 25(2).

Chen, X.X., Yu, J., Chen, J.W., 2024. A cross-sectional study of the relationship between premenstrual syndrome and missed nursing care among female nursing staff. Heliyon, 10(5).

Chen, Z., Imai, K., Zhou, X., 2023. The relationship between physical activity and premenstrual syndrome in senior high school students: a prospective study. Sci. Rep. 13(1): 5881.

Cheng, S.H., Shi, C.C., Yang, Y.K., 2013. Factors associated with premenstrual syndrome-A survey of new female university students. Kaohsiung J. Med. Sci. 29(2): 100-5.

Cheng, Z.Y., Zhang, M., Kang, Z.Z., 2003. Survey on the menstrual health of girls in Montagnard and Han Secondary Schools in Baotou city. Chin. J. Sch. Doct. 17(2): 134-5.

Fu, X.M., Zhang, J.L., Lin, X.S., 2006. Investigation and health care treatment of premenstrual syndrome in working girls. Int. Med. Health Guid. News 12(16): 142-4.

Gao, Y.P., 1999. Survey on the situation of menstrual disease among female students of Henan Academy of Finance. Chin. J. Sch. Health, (3): 236.

Hou, L.L., Wang, Z., Fei, J.F., 2019. Reliability and validity of the premenstrual symptoms screening tool in female college students. Chin. J. Clin. Psychol. (2).

Hou, L.L., Zhou, R.L., 2021. Patterns of premenstrual syndrome and depression symptoms in Chinese female university students: Results of a latent profile analysis. J. Affect. Disorders 293: 64-70.

Hsiao, M.C., Hsiao, C.C., Liu, C.Y., 2004. Premenstrual symptoms and premenstrual exacerbation in patients with psychiatric disorders. Psychiatry Clin. Neurosci. 58(2): 186-90.

Huang, D., Zhao, X.L., Zhao, M., 2018. Relationship between premenstrual syndrome and personality traits of female students in a medical college. Chin. J. Health Psychol. 26(1).

Huang, Y., Zhou, R., Wu, M., 2015. Premenstrual syndrome is associated with blunted cortisol reactivity to the TSST. Stress, 2015, 18(2): 160-8.

Hui, A.L., Bai, Z.J., 2009. Status of emmeniopathy among medical students and the impact on their study and living. Occup. Health‌‌ 25(24): 2738-9.

Jiang, J.Y., Ji, J.L., 1994. Survey and analysis of menstrual health questionnaire among 579 medical students. Chin. J. Sch. Health (04): 314-5.

health 64(2): 153-64.

Li, G.J., 2010. Analysis of the results of a survey on premenstrual syndrome among female college students. MCHC 25(1): 68-9.

Li, J.Z., Bai, J., Liu, M., 2020. Survey on the influence of personality traits，menstrual attitude and sex hormones levels on premenstrual syndrome in female college students. MCHC 35(5): 924-7.

Li, L., Lv, X.Y., Li, Y.X., 2023. Development and validation of risk prediction model for premenstrual syndrome in nurses: results from the nurses-based the TARGET cohort study. Front. Public Health 11.

Li, X.D., Lu, H., Yang, X., 2014. Study on relationship between job stress and premenstrual syndrome among Chinese nurses. Chin. J. Clin. Obstet. Gynecol. 15(5).

Li, Y.C., Jiang, J.W., Halldorsdottir, T., 2023 Premenstrual disorders and gender differences in adolescent mental health. J. Affect. Disorders 340: 930-7.

Li, Y.M., Dong, X.M., Wang, R., 2013. Survey on the occurrence of premenstrual syndrome and its influence among female university students. Prev. Med. Trib. 19(11 ).

Li, Y.M., Xiong, H.M., Liu, H., 2009. Prevalence of premenstrual syndrome in female students of higher vocational colleges and its influential factors. Health Med. Res. Prac. High Inst. 6(3): 50-2.

Liang, X.L., Yang, T., Liao, Y.T., 2024. The impact of comorbid premenstrual syndrome or premenstrual dysphoric disorder on the clinical characteristics of bipolar disorder among Han Chinese women. Arch. Womens Ment. Health 27(1): 67-75.

Liu, R., Xu, S.J., 2022. A survey on the current status and influencing factors of premenstrual syndrome among female college students in a medical school. J. Mod. Med. Health 38(S2).

Liu, X.J., 2015. Occurrence and influencing factors of premenstrual syndrome among female college students in a traditional Chinese medicine college. Chin. J. Sch. Doc. 29(6): 416-7.

Liu. S.H., Zhu, Q.Q., Tong, Y.H., 2023. Current situation of premenstrual syndrome of female college students and the investigation and analysis of coping measures. J. Women Child. Health Guide 2(8): 61-3,6.

Lu, H.M., Liu, G.X., Chen, X.Y., 2014. Analysis of the correlation between drug-taking behavior and premenstrual syndrome in women of childbearing age. J. Yangtze Univ. (Soc. Sci. Ed.) 11(11).

Ma, S.M., Rao, S.M., Tian, X.F., 2024. Correlations between traditional chinese medicine constitutions and premenstrual syndrome of female college students in Tianji. Basic Tradit. Chin. Med. 3(6).

Mao, K., Chang, A., 1985. the premenstrual-syndrome in chinese. Aust. N. Z. J. Obstet. Gynaecol. 25(2): 118-20.

Park, J., Lee, J.J., Park, S.M., 2022. Endocrine disrupting chemicals and premenstrual syndrome in female college students in east Asia: A multi-country study. Int. J. Womens Health 14: 167-77.

Qian, C., Bai, Y.Q., Zhu, X.Q., 2011. Present occurrence and related factors of premenstrual syndrome among female students in higher vocational college. Chin. Rural Health Serv. Admin. 31(12): 1295-7.

Qiao, M.Q., Zhang, H.Y., Liu, H.M., 2012. Prevalence of premenstrual syndrome and premenstrual dysphoric disorder in a population-based sample in China. Eur. J. Obstet. Gynecol. Reprod. Biol. 162(1): 83-6.

Qin, R., Mao, C., Li, G.P., 2024. Network structure of complex interactions of premenstrual syndrome and influencing factors in young adult women. J. Affect. Disorders 354: 199-205.

Shang, J.Y., Liu, Y., He, S.Q., 2022. Exploring the relationship between finger length ratio and premenstrual syndrome in female students of a high school in Guizhou province. J. Qiannan Med. Coll. Natl. 35(2).

Shen, H.C., Li, J.Z., 2018. The impact of student mental health on premenstrual syndrome. J. Mudanjiang Med. Univ. 39(2).

Shi, X.Y., Chen, M., Pan, Q., 2024. Association between dietary patterns and premenstrual disorders: a cross-sectional analysis of 1382 college students in China. Food Funct. 15(8): 4170-9.

Shi, Y.Q., Shi, M.Y., Liu, C., 2023. Associations with physical activity, sedentary behavior, and premenstrual syndrome among Chinese female college students. BMC womens health 23(1).

Shi. M., Wei, H.H., Chen, Z.L., 2021. The status quo and influencing factors of premenstrual syndrome among female college students in a medical school. J. Shenyang Med. Coll. 23(1).

Song. P.G., Wang, D.D., Wu, K.J., 2013. Correlation between premenstrual syndrome and adaptability for female college students. Chin. J. Sch. Health (3).

Tao, A.H., Liu, J., Deng, Y.Y., 2023. A correlation analysis between appetitive traits and premenstrual syndrome among female college students. Health Educ. Health Promot. 18(2): 120-3,203.

Wang, D.L., Li, X., Fan, J.Y., 2023. A study on the current situation and influencing factors of premenstrual syndrome in clinical nurses. J. Nurs. Admin. 23(5): 325-8,80.

Wang, H., Ding, M.H., Pan, A.P., 2013. Investigation of premenstrual syndrome and psychological status among female medical students in Taizhou higher vocational college. J. Chi. Med. Herald 10(35): 138-40,43.

Wang, J., Zhang, Y.A., Feng, B.X., 2021. Impact of premenstrual syndrome on coping styles in female nursing student. Chin. Sci. Technol. J. Database Med. (6).

Wang, P.P., Zhang, J.W., 2019. Investigation and analysis of the occurrence of premenstrual syndrome and related factors among female college students in a university. J. Pract. Gynecol. Endocrinol. 6(12): 105-6.

Wang, X., Ge, Y.H., Liu, Y.X., 2024. The association between occupational stress, sleep quality and premenstrual syndrome among clinical nurses. BMC Nurs. 23(1).

Wang, X.H., Wang, H.X., Deng, B.B., 2019. Premenstrual syndrome on the quality of life of medical staffs and its influencing factors. Chin. Occup. Med. 46(3).

Wang, Y.H., Lin, S.Q., Chen, R., 2012. Pattern of moderate-to-severe symptoms of premenstrual syndrome in a selected hospital in China. J. Obstet. Gynaecol. Res. 38(1): 302-9.

Wang, Y.H., Luo, Q., Ma, J., 2013. Analysis of symptoms related to premenstrual syndrome in female students of a medical college of higher education. Chin. J. Sch. Health 34(12).

Wu, X.M., Zhang, L., Jiang, X.Q., 2008. Investigation of premenstrual syndrome in reproductive women in the Fu-tian district of Shenzhen city. J. Chin. Med. Herald 5(22).

Xie, W., 2003. Prementrual stress syndrome in female secondary specialized school students. Chin. J. Health Educ. 19(10): 813-4.

Yang, L., Li, F., Lu, J.M., 2016. Premenstrual syndrome's influence on life quality of girls in a university in Xi'an. Chin. J. Woman Child Health Res. 27(5).

Yang, Y.Y., 2012. A Survey of the menstrual conditions of 2050 high school girls in Xiamen First High School. Med. Innov. Chin. 9(15): 85-6.

Yao, S.Q., Wu, Q.F., Yang, J.Y., 2009. Effect of occupational stress on menses and sex hormones of female knitting workers. Chin. J. Ind. Hyg. Occup. Dis. 27(12): 716-20.

Yu, Q., Wang, H.X., 2016. Investigation and analysis on premenstrual syndrome among female college students. Chin. J. Pract. Nurs. 32(25): 1959-62.

Yuan, Y., Wu, W.G., Liu, X.Q., 2016. Survey of premenstrual syndrome among female students at three universities in Xiamen, China. Chin. J. Sch. Health 37(4): 612-4.

Zhang, H.L., Zhu, M.X., Kong, M.R., 2012. Survey of premenstrual symptoms and premenstrual syndrome in 807 women with childbearing age in Macao. Chin. Nurs. Res. 26(9).

Zhang, H.Y., Qiao, M.Q., Dou, X.J., 2010. American college of obstetricians and gynecologists recommended diagnostic criteria for premenstrual syndrome in the applicability to a domestic multicenter population to explore. Prog. Obstet. Gynecol. 19(9): 692-4.

Zhang, J., 2012. A survey on the prevalence of premenstrual syndrome with liver dysfunction in female university students. J. Shanxi Coll. Tradit. Chin. Med. 35(1): 73-4.

Zhang, J.J., Yu, J., Liu, L., 2007. Application of present state questionnaire of premenstrual syndrome on cases selected. J. Shandong Univ. Tradit. Chin. Med. 31(5): 358-61.

Zhang, K.L., Su, J., Zheng, M., 2020. Investigation on status quo of premenstrual syndrome among female college students of Han nationality and minorities in a minority area. J. Mod. Med. Health 36(5).

Zhang, M., Liu, H., Feng, Y.L., 2019. Relationship between premenstrual syndrome during "examination month" and psychological disturbance and fatigue in female college students. J. Shenyang Med. Coll. 21(5).

Zhang, Q., Du, C.S., Liu, H.Y., 2019. Reliability and validity of Chinese version of the premenstrual coping measure scale. J. Nurs. Train. 34(13): 1175-8.

Zhang, X.Q., Wang, D.Y., 2024. The relationship between perceived stress and premenstrual syndrome The relationship between perceived stress and premenstrual syndrome. Psychol. Mag. 19(11): 92-4.

Zhang, Y., 2013, Influence of TCM constitution of female college students on premenstrual syndrome. Chin. Nurs. Res. 27(29): 3240-1.

Zhang, Y.Q., 2014. Current status of the occurrence of premenstrual syndrome among female students in a university. Chin. J. Sch. Health 35(9): 1423-5.

Zhang, Z.Z., Song, L.J., 2010. Prevalence of premenstrual syndrome and the influencing factors among female undergraduates. Chin. J. Sch. Health 31(6).

Zhao, G.L., Wang, L.H., Qu, C.Y., 1998. Prevalence of premenstrual syndrome in reproductive women and its influential factors. Chin. J. Obstet. Gynaecol. 33(4).

Zheng, H.Y., Zhang, X.Z., Wang, Z.H., 2015. Menstrual disorder factors and countermeasure analysis of nursing-major female college students in our college. J. Taishan Med. Coll. (7): 755-8.

Zhong, M., Du, G.F., Liu, H.R., 2023. Relationship between insufficient sleep and premenstrual syndrome in female college students: the chain mediating effect of negative stress perception and selfrated health status. Psychol. Mag. 18(22): 81-4.

Zhong, M.T., Lei, X.X., Yao, S.Q., 2017. The application of the shortened premenstrual assessment form in chinese female undergraduates. Chin. J. Clin. Psychol. 25(1).

Zhou, H.M., Tang, M., Zeng, L.P., 2017. Comparison of premenstrual syndrome between female students of sports and non-sports major in universities. Chin. J. Sch. Health 38(2).

Zhou, X.D., Liu, X.D., Li, J.Q., 2022. Current situation and influencing factors of premenstrual syndrome in female college students in a medical school in Xinjiang. Chin. J. Sch. Doc. 36(4).

Zhu, S.P., Wu, J.Y., Qin, Y.C., 2021. A survey of adolescent women's on knowledge, attitude and practice sexual and reproductive health and an analysis of their educational needs in Jiangmen. CHSM 12(4): 20-4.

Zou, Y.H., Wang, H.X., Tan, M.Y., 2023. Construction of a risk prediction model for premenstrual syndrome in women of childbearing age. Mod. Nurse 30(8).
